# Advancing Flexible Optoelectronic Synapses and Neurons with MXene-Integrated Polymeric Platforms

**DOI:** 10.3390/nano15191481

**Published:** 2025-09-27

**Authors:** Hongsheng Xu, Xiangyu Zeng, Akeel Qadir

**Affiliations:** 1 Industry-Education-Research Institute of Advanced Materials and Technology for Integrated Circuits, Anhui University, Hefei 230601, China; 2 Hangzhou Institute of Technology, Xidian University, Hangzhou 311200, China; zengxiangyu@xidian.edu.cn; 3 School of Information Engineering, Xi’an Eurasia University, Xi’an 710065, China; akeelqadir@eurasia.edu

**Keywords:** polymer composites, flexible electronics, optoelectronic synapses, neuromorphic computing, MXenes, brain-inspired computing

## Abstract

Neuromorphic computing, inspired by the human brain’s architecture, offers a transformative approach to overcoming the limitations of traditional von Neumann systems by enabling highly parallel, energy-efficient information processing. Among emerging materials, MXenes—a class of two-dimensional transition metal carbides and nitrides—have garnered significant attention due to their exceptional electrical conductivity, tunable surface chemistry, and mechanical flexibility. This review comprehensively examines recent advancements in MXene-based optoelectronic synapses and neurons, focusing on their structural properties, device architectures, and operational mechanisms. We emphasize synergistic electrical–optical modulation in memristive and transistor-based synaptic devices, enabling improved energy efficiency, multilevel plasticity, and fast response times. In parallel, MXene-enabled optoelectronic neurons demonstrate integrate-and-fire dynamics and spatiotemporal information integration crucial for biologically inspired neural computations. Furthermore, this review explores innovative neuromorphic hardware platforms that leverage multifunctional MXene devices to achieve programmable synaptic–neuronal switching, enhancing computational flexibility and scalability. Despite these promising developments, challenges remain in device stability, reproducibility, and large-scale integration. Addressing these gaps through advanced synthesis, defect engineering, and architectural innovation will be pivotal for realizing practical, low-power optoelectronic neuromorphic systems. This review thus provides a critical roadmap for advancing MXene-based materials and devices toward next-generation intelligent computing and adaptive sensory applications.

## 1. Introduction

The human brain, often regarded as a biological computer, exhibits exceptional energy efficiency operating on just ~20 W of power while performing complex tasks such as perception, decision-making, and adaptive learning with remarkable robustness [1]. In contrast, conventional artificial intelligence (AI) systems, though effective at specialized tasks like image classification, natural language processing, and gameplay, remain fundamentally limited. These systems are typically constrained by algorithmic rigidity and are executed on digital hardware architectures that suffer from the von Neumann bottleneck-where the separation between memory and processing units results in bandwidth limitations, latency, and elevated power consumption [2]. To overcome these barriers, neuromorphic computing (NC) has emerged as a promising paradigm inspired by the human brain’s neural architecture and dynamics [2,3]. NC architectures employ synthetic neurons and synapses to enable event-driven, parallel information processing, often implementing spike-timing-dependent plasticity (STDP) and in-memory computation [4,5,6,7]. Systems such as IBM’s TrueNorth chip-integrating one million spiking neurons and 256 million synapses-have demonstrated real-time AI performance at ultralow power (63 mW), using CMOS-based circuitry to emulate neuronal activity [8]. However, despite these breakthroughs, CMOS implementations remain challenged by integration density, dynamic plasticity limitations, and the inherent constraints of conventional semiconductor materials. Emerging device concepts based on memristors [9,10,11,12,13,14], advanced transistors [15,16,17,18], phase-change materials [19,20], magnetoresistive and ferroelectric elements [21,22,23,24,25,26] have shown promise in emulating neural and synaptic behaviors with high integration potential. Yet, most electronic neuromorphic systems are still bound by interconnect latency and energy inefficiencies, especially as the scale of sensory data and computation grows [27].

Optoelectronic neuromorphic systems have gained considerable traction due to their ability to combine the advantages of photonic and fle computation. By using light as an input and/or modulation signal, these systems benefit from superior bandwidth, low signal interference, reduced power consumption, and enhanced processing speeds [28,29,30,31]. For example, optoelectronic synapses have been demonstrated with energy consumption as low as 66 fJ per spike [32], and neuromorphic visual systems using photodiode arrays have achieved real-time optical encoding at speeds of 20 million bins per second [33]. Optoelectronic neurons, functioning as photodetectors and leaky integrators, can produce spikes when integrated optical signals exceed a threshold [34,35], mimicking biologically relevant dynamics like leaky integrate-and-fire (LIF) and threshold-triggered spiking [36,37,38,39,40,41].

The first optoelectronic artificial neuron was demonstrated as early as 1989, utilizing optical interconnections in a Hopfield network [42]. Since then, a variety of device architectures have emerged-ranging from optically gated carbon nanotube synapses [43], to single-transistor photoneurons [44], and all-optically controlled (AOC) bilayer IGZO synapses exhibiting long-term plasticity [45,46,47,48,49,50,51,52,53]. These devices offer integrated sensing-computing-memory (ISCM) capabilities, making them particularly attractive for edge AI, real-time machine vision, and brain-inspired perception systems. Yet, despite these advances, performance, scalability, and material compatibility continue to pose significant challenges-especially in the context of flexible, biocompatible, and wearable electronics.

In parallel, the field of flexible electronics has increasingly relied on polymeric materials as the foundational substrate and active component. Polymers such as polyimide (PI), polyethylene terephthalate (PET), polydimethylsiloxane (PDMS), and polyvinyl alcohol (PVA) offer unparalleled advantages including mechanical flexibility, low-cost solution processability, lightweight nature, and biocompatibility [54,55]. For neuromorphic systems targeting wearable and implantable applications, the integration of functional nanomaterials with these polymeric platforms is not just beneficial but essential. The polymer matrix does more than provide passive support; it actively influences device performance through its chemical interactions with the nanomaterial, its role in modulating ion transport, and its determination of the overall mechanical robustness of the system [56,57].

This has motivated intense exploration of two-dimensional (2D) materials, with MXenes standing out as a particularly compelling class. Discovered in 2011, MXenes are a family of 2D transition metal carbides and nitrides that combine exceptional electrical conductivity [58], high hydrophilicity [59], tunable surface chemistry, biocompatibility, optical responsiveness [60], and excellent mechanical flexibility [61]. These properties make MXenes especially well-suited for neuromorphic optoelectronic systems, particularly in flexible or implantable applications. Beyond their success in energy storage [62], sensing [63], and catalysis [64,65], MXene-based memristors and transistor devices have demonstrated large ON/OFF ratios, low switching voltages, and emulation of various forms of synaptic plasticity [66]. Recent MXene-based neuromorphic devices have achieved high recognition accuracy, robust endurance, and energy-efficient performance, showing strong potential for use in high-density memory, in-memory computing, and intelligent vision systems. Their inherent compatibility with flexible substrates and their ability to support hybrid optical–electrical functionality further enhance their appeal.

This review provides a comprehensive analysis of MXene-based neuromorphic optoelectronic systems, beginning with an overview of neuromorphic computing and optoelectronic device mechanisms. We then introduce the synthesis, structure, and key properties of MXenes relevant to this field. Finally, we discuss the most recent innovations in MXene-based artificial synapses and neurons, and outline challenges and future directions for realizing next-generation, brain-inspired computing architectures.

## 2. Fundamentals of Neuromorphic Computing Systems

**Figure 1:**
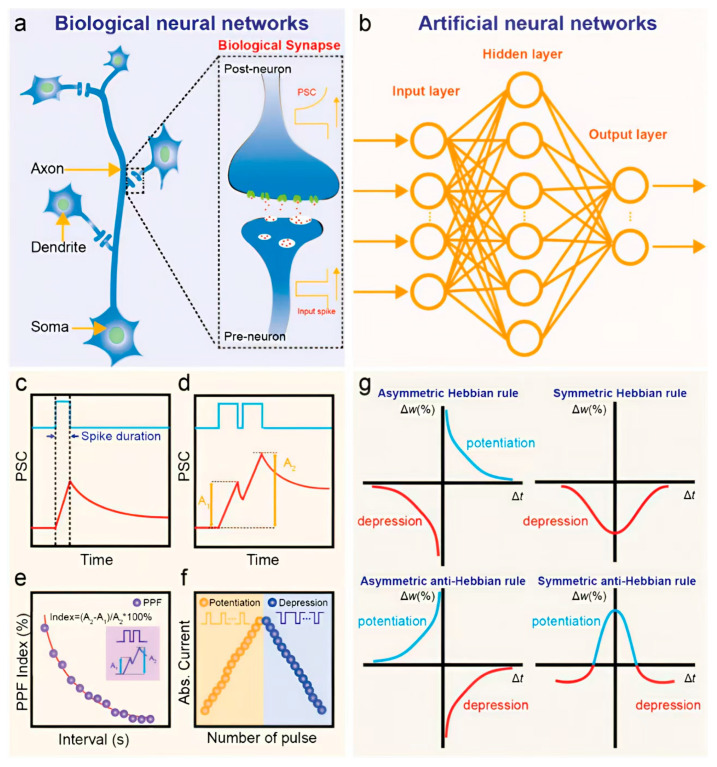
Conceptual overview of biological and artificial neural networks, along with simulated synaptic behaviors. (**a**) Schematic of a biological neural network (BNN), with an inset showing a detailed view of a biological synapse consisting of pre- and postsynaptic components. (**b**) Basic architecture of an artificial neural network (ANN), comprising input, hidden, and output layers. (**c**–**g**) Simulated curves representing key synaptic functions in neuromorphic systems: (**c**) excitatory postsynaptic current (EPSC); (**d**) paired-pulse facilitation (PPF); (**e**) PPF index as a function of inter-spike interval; (**f**) long-term potentiation (LTP) and depression (LTD); (**g**) four types of spike-timing-dependent plasticity (STDP), illustrating asymmetric/symmetric Hebbian and anti-Hebbian learning rules [77].

Following the growing limitations of conventional digital computing architectures, neuromorphic systems have gained significant attention as an alternative approach to realizing brain-inspired intelligence. These hardware-based systems are designed to emulate the structure and function of artificial neural networks (ANNs), offering real-time learning capabilities with reduced latency and energy consumption compared to traditional von Neumann-based processors [67]. Such characteristics make neuromorphic systems particularly attractive for data-intensive AI tasks like image recognition and adaptive control. Biologically, the human brain consists of approximately 10^11^ neurons interconnected by an estimated 10^15^ synapses. These synapses form dynamic communication links that not only transmit electrical signals between neurons but also modulate the strength of those signals through processes collectively termed synaptic plasticity, the core mechanism underlying learning and memory. As illustrated in Figure 1a, a synapse connects the axon of one neuron to the dendrite of another, forming a network capable of highly complex and parallel information processing.

Neuromorphic hardware seeks to replicate these brain-like behaviors using emerging electronic components such as memristors and transistors, which act as analogs to biological synapses and neurons [68]. In ANNs, as depicted in Figure 1b, artificial neurons are arranged in interconnected layers where the synaptic weights (SWs) define the strength of connections between units. These weights are represented in hardware by the conductance states of neuromorphic devices, enabling adaptive, nonlinear learning and self-organization in response to input stimuli [69]. In experimental setups, synaptic plasticity-defined as the tunable SW in response to external input [70]-is typically evaluated by measuring the postsynaptic current (PSC) generated in response to electrical or optical pulses (Figure 1c). A single presynaptic stimulus can induce an excitatory postsynaptic current (EPSC) or, in some cases, an inhibitory PSC (IPSC) [71], which collectively determine the nature of the neural signal propagation. Plasticity is typically categorized as either short-term (STP) or long-term plasticity (LTP) depending on the duration of synaptic modification, which can range from milliseconds to several days. In paired-pulse protocols (Figure 1d), a facilitation effect-where the second response is stronger than the first-is termed paired-pulse facilitation (PPF), while a diminished second response is called paired-pulse depression (PPD). The inter-spike interval (Δt) between two pulses critically influences the PPF index, with longer intervals generally reducing facilitation, as shown in Figure 1e [72].

Through controlled modulation of stimulus parameters-including pulse amplitude, frequency, duration, and number-short-term responses can transition into long-term adaptations. For instance, repeated positive stimuli can enhance synaptic strength (long-term potentiation), whereas repeated negative stimuli can weaken it (long-term depression), as illustrated in Figure 1f. These mechanisms collectively mimic how biological synapses encode learning experiences through cumulative reinforcement or inhibition. At a more advanced level, higher-order learning rules, such as Hebbian learning (“cells that fire together wire together”) govern how neural connections adapt based on correlated activity [73]. Among these, spike-timing-dependent plasticity (STDP) and spike-rate-dependent plasticity (SRDP) are especially prominent [74]. STDP adjusts SWs depending on the precise timing between pre- and postsynaptic spikes, often producing characteristic weight change curves (Δw) as a function of Δt, as seen in Figure 1g [75]. Meanwhile, SRDP modulates learning based on the frequency of presynaptic firing-where higher spike rates typically lead to potentiation and lower rates to depression [76].

Together, these mechanisms form the foundational principles of NC systems. As the field advances, translating these brain-like operations into practical device-level implementations depends critically on material innovation and functional integration. In this context, the choice of material directly affects synaptic fidelity, energy efficiency, optical response, and scalability. In the following section, we introduce MXenes that has emerged as a highly promising material platform for neuromorphic optoelectronics. We begin by exploring their structural features, synthesis methods, and intrinsic optical and electrical properties, which lay the groundwork for their integration into high-performance neuromorphic synapses and neurons. This understanding is essential for appreciating the capabilities and design strategies of MXene-based optoelectronic neuromorphic systems, which we examine in detail in the subsequent sections.

## 3. Fundamentals of MXenes

MXenes are a class of 2D materials with atomic-scale thickness that possess several advantages, including a planar nanosheet structure, excellent electrical conductivity, and remarkable flexibility. These properties make MXenes highly suitable for applications as flexible semiconductor layers and electrodes. The diverse surface terminating functional groups present on MXenes provide various functionalities such as charge trapping and catalytic activity. Furthermore, MXenes’ compatibility with other 2D materials facilitates the development of multifunctional devices with enhanced performance. This section discusses the material structures, and unique properties of MXenes.

### 3.1. Structures

**Figure 2:**
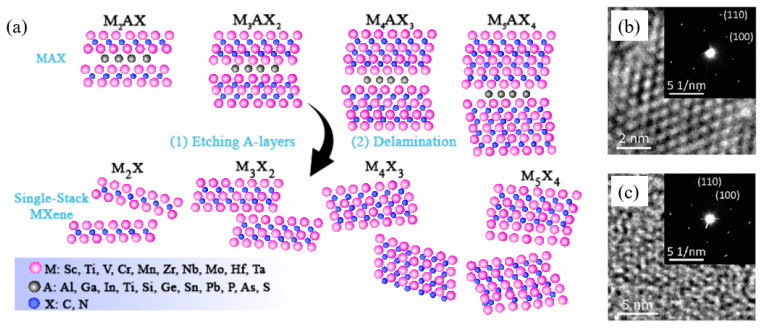
Schematic and characterization of MXenes. (**a**) Synthesis of four kinds of typical MXenes (M2X, M3X2, M4X3, and M5X4) from MAX phases through etching and delamination processes. (**b**,**c**) HRTEM images of Nb2AlC MAX phase (**b**) and Nb2C MXene flake (**c**) (inset: corresponding SAED patterns) [85].

The general chemical formula of MXenes is M_n+1_X_n_T_x_ (where n = 1, 2, 3, or 4), where M represents one or two early transition metals such as Ti, Cr, or W; X denotes carbon and/or nitrogen atoms; and T_x_ represents surface functional groups typically including –O, –F, –Cl, and –OH [78]. As illustrated in Figure 2a, MXenes are derived from their parent compounds known as MAX phases, which have the general formula M_n+1_AX_n_ (n = 1, 2, 3, or 4). In MAX phases, A mainly consists of group IIIA or IVA elements such as Al or Si. The X atoms occupy octahedral sites between M layers, while the A layer is interspersed within the M_n+1_X_n_ layers. The bonding between M and A atoms is purely metallic, whereas the M–X bond exhibits both covalent and metallic bonding characteristics [79,80,81,82]. MXenes are produced by selectively etching away the A layers from the MAX phases. MAX phases typically crystallize in layered hexagonal structures with symmetry belonging to the space group P63/mmc, as confirmed by high-resolution transmission electron microscopy (HRTEM) and selected area electron diffraction (SAED), shown in Figure 2b [83]. Upon removal of the A layers, the resulting 2D layered MXenes retain the arrangement of M and X atoms similar to that of the MAX phases (Figure 2c) [84].

The diverse composition, tunable surface chemistry, and bulk properties of MXenes impart unique and valuable characteristics, making them promising candidates for advanced flexible materials used in memory and neuromorphic devices [86,87]. The following sub-sections provide an overview of MXene properties, including their electrical, and optical characteristics.

### 3.2. Electrical Properties

The electrical behavior of MXenes is strongly influenced by their material structure, bonding nature, surface terminations, and defect states [88]. Due to variations in composition and surface functionalization, MXenes can exhibit metallic, semiconducting, or even topological insulating behavior. For example, Ti_3_C_2_T_x_ MXene demonstrates excellent conductivity (greater than 2.40 × 10^4^ S cm^−1^), primarily attributed to the combined effects of surface functional groups on the nanosheets and the electron contribution from Ti atoms, establishing it as a strong candidate for electronic applications [58,89].

Density Functional Theory (DFT) calculations show that pristine MXenes without terminal groups generally behave as metals, characterized by the overlap of conduction bands (CBs) and valence bands (VBs) at the Fermi level [90]. However, the presence of abundant surface terminations can lower the Fermi level below the d orbital of M atoms, inducing a band gap and transforming the MXene into a semiconductor. Despite this, most M_n+1_X_n_T_x_ MXenes (such as Ti_3_C_2_T_x_) maintain metallic properties, especially when the number of layers (n) is large, regardless of surface termination [91]. In monolayer MXenes, electronic structures can be tuned by adjusting surface functional groups and the associated geometric conformations [92]. For multilayer MXenes, intercalation of agents between layers increases resistance by expanding the interlayer spacing [93]. Additional advantageous electrical properties of MXenes include high carrier mobility (~1 cm^2^ V^−1^ s^−1^), high charge carrier density (~3.8 × 10^22^ cm^−3^), and a tunable work function controlled through surface chemistry modifications [94,95,96]. Utilizing the high conductivity and nanoscale carrier transport channel length of MXenes, synaptic transistors with MXene electrodes can achieve linear conductance tuning, enhancing their performance in neuromorphic applications.

### 3.3. Optical Properties

MXenes exhibit excellent electronic transport properties and remarkable optical characteristics spanning from the ultraviolet (UV) to near-infrared (NIR) ranges, making them highly suitable for optical applications such as optoelectronic memristors [97]. Their optical features include outstanding transparency, a strong photothermal effect, and notable plasmonic effects. For instance, 5 nm-thick Ti_3_C_2_T_x_ films can achieve a transmittance as high as 91.2%, with significant light absorption in the 300–500 nm wavelength range, which is crucial for transparent conductive electrodes and other optoelectronic devices [98]. The optical properties of MXenes are strongly influenced by their surface functional groups and chemical composition [71]. DFT calculations reveal that in the UV region, all surface terminations enhance the absorption and reflectivity of Ti_3_C_2_T_x_ MXenes. However, in the visible range, F- or OH-terminated Ti_3_C_2_T_x_ show lower absorption and reflectivity compared to pristine MXene [99].

Elemental substitutions at the M and X sites also affect optical behavior. For example, partially substituting carbon with nitrogen in Ti_3_AlC_2_ MAX phases creates Ti_3_CNT_x_ MXenes, which display a blue shift in their main absorption peak relative to Ti_3_C_2_T_x_ [97]. Additionally, doping with magnetic transition metals such as Fe, Co, and Ni increases the optical absorption coefficient of Ti_3_C_2_ across UV, visible, and NIR regions, likely due to modifications in the electronic structure induced by these magnetic atoms.

Some MXene materials also exhibit electrochromic behavior, where their optical absorption can be modulated by an applied electric field. This property makes them promising candidates for applications in smart windows, display screens, and other devices requiring tunable transparency or color change [100]. Furthermore, due to the tendency of MXenes to undergo irreversible oxidation, previous studies have demonstrated that partially oxidized Ti_3_C_2_T_x_ can generate photocurrent under visible light pulse stimulation [101]. This effect enables MXenes to simulate visual image processing in brain-inspired devices, highlighting their potential in neuromorphic optoelectronics.

Building on the foundational understanding of MXene structures, synthesis techniques, and their unique optical and electrical properties, significant progress has been made in leveraging these materials for the development of optoelectronic synaptic devices. These neuromorphic components are designed to emulate biological synapses using light as the primary stimulus, offering distinct advantages in terms of ultrafast response, parallelism, and energy efficiency. Recent studies have demonstrated a wide variety of MXene-based optoelectronic synapses, where different physical mechanisms-including photogating, photovoltaic effects, photothermal conversion, and charge trapping-are employed to modulate SWs and reproduce key features such as plasticity, learning, and memory. In the following section, we provide a comprehensive overview of these research efforts, categorized by the underlying physical principles governing their operation, to highlight the versatility and potential of MXene-enabled neuromorphic optoelectronics.

### 3.4. MXene-Polymer Composites for Flexible Neuromorphic Devices

The integration of MXenes into polymeric matrices is a critical strategy for harnessing their exceptional properties in practical, flexible devices. This synergy creates advanced composites where the polymer host enhances processability, mechanical stability, and environmental resilience, while the MXene filler provides high electrical conductivity, rich surface chemistry, and functional active sites for neuromorphic operation [102]. Common fabrication methods are designed to achieve a homogeneous dispersion of MXene nanosheets within the polymer, which is paramount for optimal performance. Solution blending is the most prevalent technique, where aqueous MXene dispersions are mixed with polymer solutions (e.g., PVA, PEDOT:PSS) and cast into films [87]. More advanced methods like in situ polymerization, where monomers (e.g., pyrrole, aniline) are polymerized in the presence of MXenes, lead to a more uniform distribution and stronger interfacial interactions, minimizing MXene re-stacking and maximizing the functional interface area [103]. Layer-by-layer (LbL) assembly has also emerged as a powerful technique to precisely control the nano-architecture of MXene-polymer multilayers, enabling fine-tuning of electrical and mechanical properties [104].

The interfacial compatibility between MXenes and polymers is a key determinant of composite performance. This is often driven by non-covalent interactions, such as hydrogen bonding between the hydroxyl (–OH) or terminal oxygen groups of MXenes (e.g., Ti_3_C_2_T_x_) and functional groups (e.g., –OH in PVA, carbonyls in polyurethanes) on polymer chains [105]. In some cases, electrostatic interactions or van der Waals forces also play a significant role. This strong interfacial adhesion is crucial for two primary reasons: it effectively prevents the re-stacking of MXene sheets, preserving their high active surface area, and it facilitates efficient charge transfer across the interface, which is vital for electronic and ionic conduction in synaptic devices [82]. The resulting composites exhibit a unique combination of properties. They retain the high electrical conductivity of the MXene network (often exceeding 1000 S/cm even at low loading levels) while inheriting the superior mechanical flexibility and toughness of the polymer host [61]. For instance, MXene/PVA nanocomposite films have been reported to withstand tensile strains exceeding 10% without significant electrical performance degradation, making them suitable for repeatedly bendable and stretchable electronics [106]. Furthermore, the polymer matrix acts as a protective barrier layer, mitigating the oxidative degradation of MXenes that typically occurs in humid or aqueous environments. Studies have shown that encapsulating MXenes in a polymer like PDMS or epoxy can significantly enhance long-term stability, retaining conductive performance for weeks instead of days under ambient conditions [107]. In neuromorphic applications, the choice of polymer is not merely passive. It directly governs device physics: ion-conducting polymers (e.g., polyethylene oxide (PEO)) modulate cation (e.g., Li^+^, H^+^) migration kinetics a key mechanism for simulating synaptic weight updates in electrolyte-gated transistors. Meanwhile, the dielectric properties of polymers influence charge trapping and de-trapping dynamics at the MXene-polymer interface, enabling the emulation of short-term and long-term plasticity. Therefore, the rational design of the MXene-polymer interface through polymer selection, functionalization, and processing is a central theme in the development of high-performance, reliable, and flexible optoelectronic synapses and neurons [108,109,110].

## 4. MXene-Based Optoelectronic Synapses

### 4.1. MXene/VP Synaptic Heterojunction

**Figure 3:**
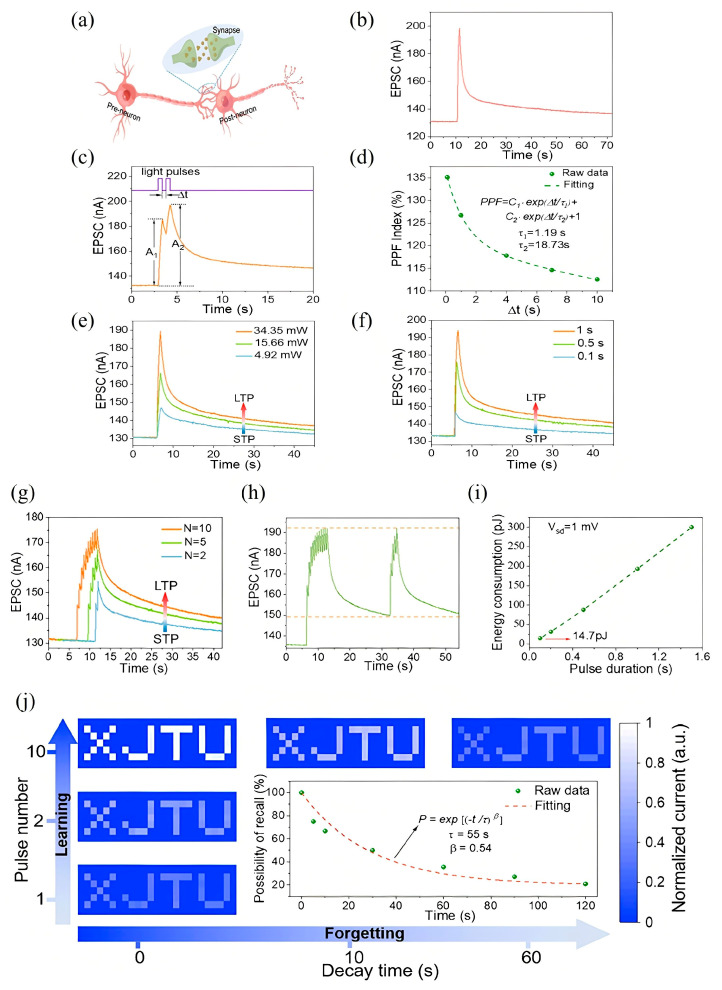
(**a**) Schematic illustration of biological neurons and synapses. (**b**) Excitatory postsynaptic current (EPSC) response of the MXene/VP optoelectronic synapse triggered by a single light pulse (360 nm wavelength, 1 s duration). (**c**) EPSC response elicited by paired light pulses with varying inter-pulse intervals (Δt). (**d**) Paired-pulse facilitation (PPF) index as a function of the interval time between light pulse pairs, demonstrating short-term plasticity dynamics. (**e**,**f**) Transition from short-term potentiation (STP) to long-term potentiation (LTP) induced by increasing the light pulse intensity (**e**) and pulse duration (**f**), respectively. (**g**) STP-to-LTP transition achieved by increasing the number of consecutive light pulses. (**h**) Demonstration of “learning-experience” behavior in the MXene/VP optoelectronic synapse, reflecting adaptive synaptic plasticity. (**i**) Energy consumption profile of the MXene/VP optoelectronic synapse during operation achieving an ultra-low energy consumption of 14.7 pJ (at a 0.1 s pulse width and 1 mV bias). (**j**) Normalized EPSC mapping images forming the “XJTU” letter pattern under increasing numbers of light pulses (vertical axis) and decay time progression (horizontal axis). Inset: The synaptic decay process fitted with the Ebbinghaus forgetting curve, illustrating memory retention characteristics [123].

In recent advancements, various MXene-based optoelectronic synaptic devices have been developed, leveraging diverse physical mechanisms to emulate key features of biological learning and memory. Among these, the MXene/vanadium pentoxide (VP) heterojunction serves as a representative case, demonstrating persistent photoconductivity (PPC) that enables rich synaptic behavior under light stimulation. As schematically depicted in Figure 3a, biological synapses enable signal transmission through chemical or electrical means between neurons [111]. In this MXene/VP system, UV light pulses function as presynaptic stimuli, while the resulting channel current represents the EPSC, a proxy for SW. A single UV pulse at a low 1 mV bias triggers a measurable EPSC with bi-phasic decay (Figure 3b), maintaining a residual response (2.4% above baseline after 100 s) that closely mimics biological postsynaptic potential decay [112].

Temporal learning behaviors are also well-emulated. For instance, when two light pulses are applied in quick succession, the second EPSC (A_2_) exceeds the first (A_1_), characterized by a PPF index (A_2_/A_1_ × 100%)-a hallmark of STP essential for temporal information processing (Figure 3c) [113]. As shown in Figure 3d, this PPF index decreases from 135% to 112% as the Δt increases from 0.1 to 10 s, with decay fitting a double-exponential relaxation model (τ_1_ = 1.19 s, τ_2_ = 18.73 s), mirroring biological analogs [114]. Moreover, synaptic plasticity [115], a cornerstone of neuromorphic computing, is demonstrated through parameter tuning. As light intensity or duration increases (from 4.92 to 34.35 mW and 0.1 to 1 s, respectively), the EPSC amplitude and retention are enhanced, marking a transition from STP to LTP (Figure 3e,f) [116]. Similarly, increasing the number of light pulses also induces LTP-like behavior, analogous to synaptic strengthening through repeated stimulation in biological circuits (Figure 3g).

A notable feature of this MXene/VP device is its emulation of experience-dependent learning. During initial exposure (ten light pulses), EPSC gradually rises and decays with ~25% retention after 20 s. Remarkably, during relearning, only four pulses suffice to restore EPSC to its prior maximum, indicating memory consolidation-a hallmark of human cognition (Figure 3h) [117]. Energy efficiency, a critical metric for neuromorphic systems, was also analyzed. The device achieves ultra-low energy consumption, with a minimum of 14.7 pJ at 0.1 s pulse width under 1 mV bias, outperforming many existing 2D optoelectronic synaptic platforms (Figure 3i) [118,119,120,121]. To explore neuromorphic vision applications, a 7 × 25 pixel “XJTU” pattern was projected via a single-pixel imaging setup. The normalized EPSC grayscale represented visual memory strength, with the number of light pulses indicating repetition. After ten training cycles, the pattern became distinguishable, then gradually faded during forgetting, closely following the Ebbinghaus forgetting curve (Figure 3j). Fitting the decay yielded τ = 55 and β = 0.54, confirming brain-like memory dynamics [122].

This comprehensive behavior confirms that the MXene/VP optoelectronic synapse not only exhibits biologically relevant plasticity and learning features but also aligns well with the requirements for vision-based NC systems.

### 4.2. Decorated MXene–TiO_2_ Optoelectronic Synapse

**Figure 4:**
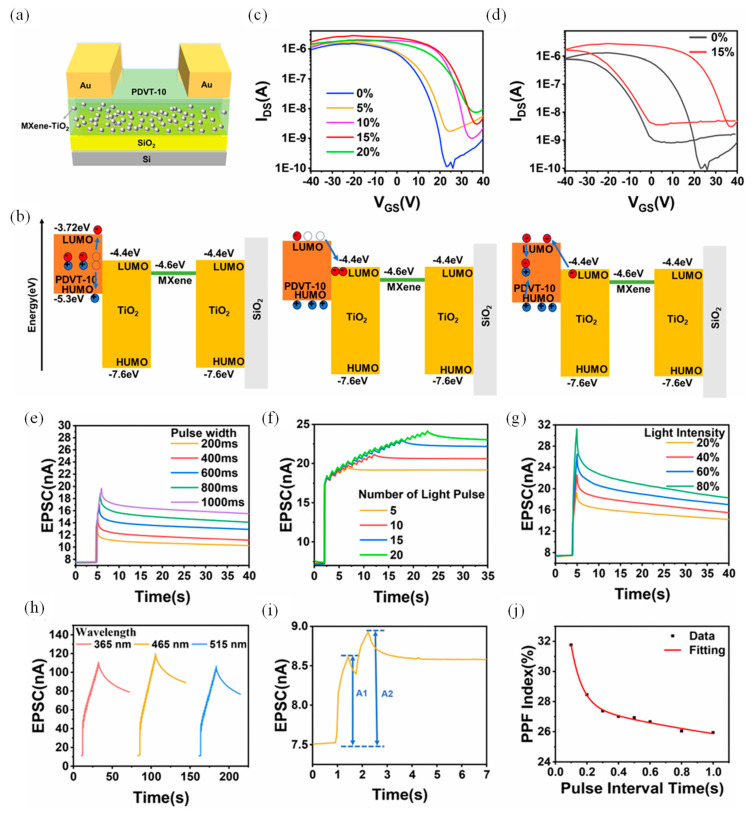
(**a**) Schematic illustration of the synaptic device structure. (**b**) Energy band alignment diagram depicting the electronic interactions within the device. (**c**) Transfer characteristics of devices with varying MXene–TiO_2_ doping ratios. (**d**) Double-sweep transfer curves for undoped (0%) and 15% doped devices, indicating Clockwise hysteresis with ΔVth = 2.1 V (15% MXene–TiO_2_) vs. 0.3 V (0% doping), enabling >10^4^ write/erase cycles linearly enhances EPSC with <8% conductance drift (**e**) Excitatory postsynaptic currents (EPSCs) induced by light pulses of varying pulse widths (465 nm wavelength, 500 ms pulse interval). (**f**) EPSCs in response to different numbers of light pulses (465 nm wavelength, 500 ms pulse width and interval). (**g**) EPSCs modulated by varying light intensities from 20% to 80% under fixed pulse width and interval conditions (465 nm wavelength). (**h**) EPSC responses to multiple light pulses of different wavelengths, demonstrating wavelength-dependent behavior. (**i**) Schematic representation of the dual-pulse facilitation mechanism. (**j**) Paired-pulse facilitation (PPF) index as a function of the interval between pulses, illustrating short-term synaptic plasticity [130].

Among the emerging strategies for neuromorphic device engineering, blending semiconducting polymers with MXene derivatives offers a unique path for achieving optically tunable synaptic behaviors. A representative implementation involves a bottom-gate top-contact optoelectronic synaptic transistor (PMOST) in which the organic semiconductor PDVT-10 is blended with oxidized MXene, forming TiO_2_ nanoparticles upon controlled oxidation (Figure 4a). The embedded TiO_2_ phase plays a dual role enabling photogating and acting as a reservoir for photogenerated charge carriers due to its rich oxygen-vacancy defect states. The working principle of the device is schematically illustrated in Figure 4b. Under optical excitation, electron-hole pairs are generated within PDVT-10. Electrons become trapped at the defect-rich TiO_2_ sites, while holes accumulate in the channel, modulating the channel conductivity. Upon cessation of illumination, the delayed release and recombination of trapped electrons with free holes in PDVT-10 govern the synaptic decay kinetics, emulating the memory retention characteristics observed in biological synapses. [124,125,126].

To assess the doping influence, transfer characteristics were recorded for varying MXene–TiO_2_ concentrations. As shown in Figure 4c, a 15% doping ratio yields the most pronounced threshold voltage shift, highlighting optimal charge modulation. The corresponding dual-sweep transfer curves in Figure 4d further validate the non-volatile behavior (Clockwise hysteresis with ΔVth = 2.1 V (15% MXene–TiO_2_) vs. 0.3 V (0% doping), enabling > 10^4^ write/erase cycles with <8% conductance drift) imparted by the MXene–TiO_2_ blend. This device platform faithfully captures the short-term memory (STM) and long-term memory (LTM) dynamics akin to biological systems. STM, characterized by transient potentiation of synaptic strength, transitions to LTM upon prolonged or repeated stimulation [127,128]. Optical pulses at 465 nm with increasing pulse durations (200–1000 ms) lead to extended retention times (Figure 4e), demonstrating duration-dependent synaptic potentiation. Additionally, the number of optical pulses significantly influences the transition: as shown in Figure 4f, 5–20 pulses induce a progressive shift from STM to LTM, enabling persistent modulation of EPSC. Figure 4g underscores the intensity dependence of synaptic strength. EPSC increases linearly as intensity rises from 20% to 80% adhering to Hebbian learning principles, that stronger stimuli yield stronger synaptic connections. The PMOST architecture also demonstrates robust pattern learning and retention through array-level implementation. An “L”-shaped photomask pattern was optically encoded on a 3 × 3 device array using 465 nm light. Following a single exposure, the optical imprint faded within 1000 s—an STM-like response. However, repeated irradiation (20 times) facilitated LTM encoding, with the “L” pattern retained beyond 1000 s post-illumination—an effective demonstration of visual memory retention in a neuromorphic framework. Multi-wavelength sensitivity is another notable feature. Figure 4h reveals differential EPSC responses to 365 nm, 465 nm, and 515 nm stimuli. The strongest EPSC occurs at 465 nm, consistent with the absorption peak of the PDVT-10/MXene–TiO_2_ composite (~450 nm), while comparable but reduced responses are observed at 365 and 515 nm due to lower absorption coefficients.

Furthermore, PPF is well-emulated in this device. As illustrated in Figure 4i, the PPF index is defined by the relative enhancement of the second EPSC compared to the first, and follows a characteristic decay with increasing Δt. The behavior is well-fitted by a dual-exponential function (Figure 4j), yielding τ_1_ = 0.06961 s and τ_2_ = 2.2206 s, reflective of distinct fast and slow synaptic relaxation pathways. This behavior is attributed to sequential electron trapping and release dynamics within TiO_2_, modulating photoconductive gain. The PMOST further exhibits a complete “learning–forgetting–relearning” cycle. Upon initial stimulation (30 light pulses), EPSC increases, representing synaptic potentiation. This is followed by a decay during the stimulus-free phase (forgetting), and a renewed EPSC enhancement upon re-stimulation (relearning). Interestingly, the relearned memory decays more slowly, indicating memory consolidation—a phenomenon also observed in biological circuits [129]. Altogether, this MXene–TiO_2_–PDVT-10 based optoelectronic synapse demonstrates a robust neuromorphic response, encompassing STM-LTM transition, multispectral sensitivity, synaptic plasticity, and memory retention, offering a biomimetic framework for future photonic neuromorphic systems.

### 4.3. MXene-Based Floating Gate Optoelectronic Synapse

**Figure 5:**
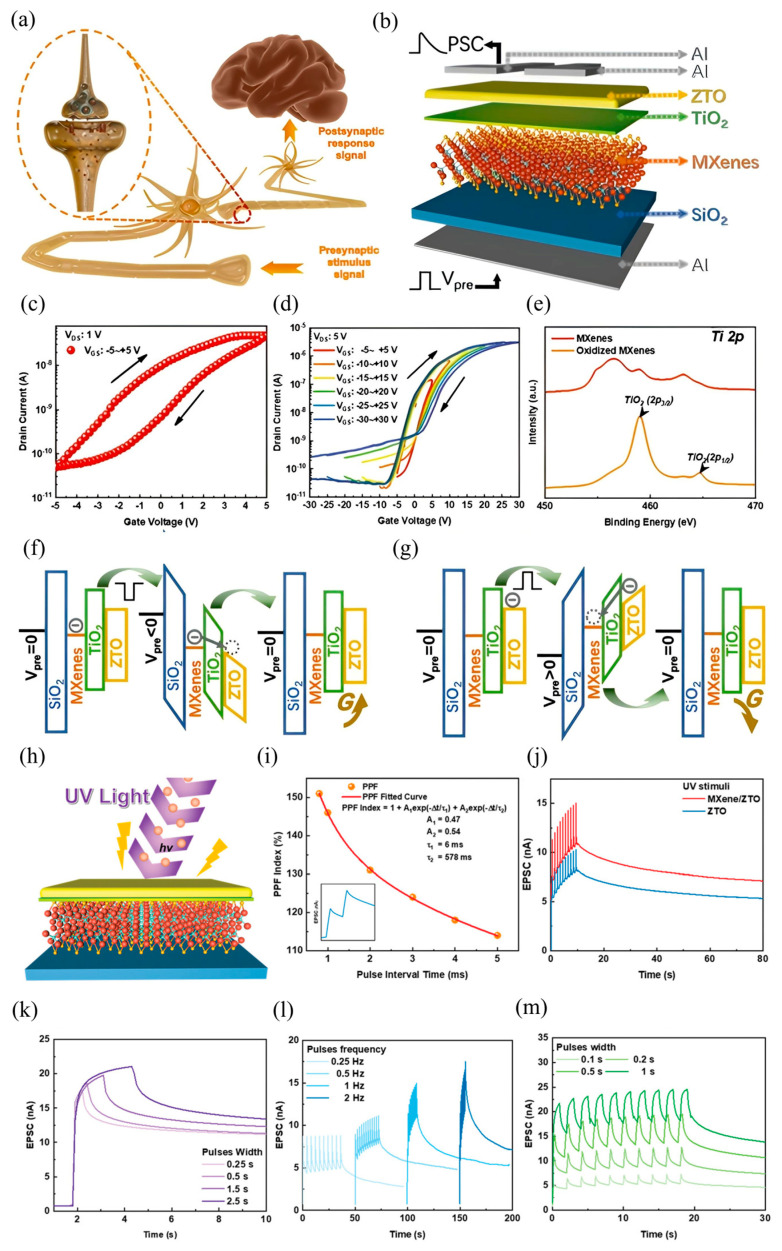
(**a**) Schematic illustration of synaptic transmission under action potentials in a biological neural system. (**b**) Structural diagram of the MXene-based field-effect transistor (MXFGT). (**c**) Transfer characteristics of the MXFGT measured at a drain-source voltage (V_DS_) of 1 V. (**d**) Transfer curves of the MXFGT under varying V_DS_ ranging from –5 V to +5 V and –30 V to +30 V. (**e**) Ti 2p X-ray photoelectron spectroscopy (XPS) spectra of pristine MXene and oxidized MXene thin films, highlighting surface chemical states. Schematic energy band diagrams of the MXFGT under a pre-applied voltage (V_pre_) with (**f**) negative gate bias and (**g**) positive gate bias conditions. (**h**) Device structure schematic of the MXFGT under ultraviolet (UV) illumination (365 nm, 3.2 mW∙cm^−2^). (**i**) Paired-pulse facilitation (PPF) index of the MXFGT under UV stimulation, demonstrating synaptic plasticity. (**j**) EPSC behavior comparison between the ZTO transistor (control group) and the MXFGT under UV stimuli, (**k**) EPSC responses of the MXFGT under single UV pulse stimuli with varying pulse widths. EPSC modulation of the MXFGT under UV pulse stimuli with varying (**l**) pulse frequencies and (**m**) pulse widths, illustrating dynamic synaptic behavior [140].

Among various biological subsystems, the nervous system plays a central role in maintaining body homeostasis by processing and transmitting information across an intricate network of neurons. As illustrated in Figure 5a, neurons receive external stimuli, convert them into electrical signals, and relay the processed information to the brain. Synapses act as crucial connectors between neurons, where neurotransmitters mediate signal transfer from the presynaptic to the postsynaptic neuron, facilitating a complex bioelectrochemical conversion. This communication strength, known as synaptic weight, underpins learning and memory processes in living systems [131,132,133]. Inspired by this biological paradigm, a synaptic MXene-based floating gate transistor (MXFGT) has been developed, as schematically shown in Figure 5b. This device integrates an Al/ZTO/SiO_2_/MXene/TiO_2_ architecture, where the MXene layer functions as the floating gate, and a self-assembled TiO_2_ tunneling barrier is formed via controlled partial oxidation of the MXenes. In this configuration, electrical spikes applied to the gate emulate presynaptic stimuli, while the resultant drain-source current (IDS) corresponds to postsynaptic currents, thus replicating EPSC-like behavior. For applications requiring flexibility, this floating gate structure could be transferred onto an elastomeric substrate such as polydimethylsiloxane (PDMS). PDMS would provide conformability and mechanical resilience, enabling the device to function reliably on curved or moving surfaces, which is essential for wearable neuromorphic systems [55].

The electrical behavior of MXFGT is characterized by a pronounced clockwise hysteresis in the transfer curves (Figure 5c), attributed to charge storage in the MXene-TiO_2_ structure. Control devices-either pristine ZTO or MXene-doped ZTO without a floating gate-do not exhibit such hysteresis, validating the role of the MXene layer. With increasing VGS scan range (Figure 5d), a progressive negative shift in threshold voltage is observed, signifying enhanced electron trapping in the floating gate and modulation of channel conductivity via charge tunneling. Material characterizations provide further insight into the working principles. XPS spectra of Ti 2p core levels (Figure 5e) confirm the formation of TiO_2_ in oxidized MXene films, with characteristic peaks at 458.4 eV and 464.5 eV [134,135]. XRD analysis reveals both amorphous ZTO and partially crystalline TiO_2_ structures, with UV–Vis absorbance data showing enhanced optical response in the ZTO/MXene heterostructure, confirming its photoreactivity and potential for visual perception systems.

The working mechanism of MXFGT is described in the energy band diagrams (Figure 5f,g) under negative and positive Vpre. At rest, electrons are trapped in the MXene layer due to energy barriers presented by SiO_2_ and TiO_2_. Under negative gate bias, electrons tunnel into the ZTO channel, increasing its conductivity (excitatory behavior), while a positive gate pulse expels electrons back into the gate stack, decreasing conductance (inhibitory behavior). This bidirectional conductance modulation enables “write” and “erase” operations, functionally mimicking synaptic excitation and inhibition [136,137]. In addition to electrical modulation, optoelectronic performance is critical for neuromorphic applications involving spatiotemporal visual processing [121,124,132]. The MXFGT was subjected to UV illumination (365 nm), with light pulses inducing measurable EPSC responses (Figure 5h). The PPF was clearly observed (Figure 5i), showing biologically analogous plasticity with extracted time constants τ_1_ = 6 ms and τ_2_ = 578 ms, reflecting sustained synaptic retention under optical excitation.

The enhanced UV-induced EPSC is attributed to two synergistic mechanisms: (i) generation of photoexcited carriers through oxygen vacancy ionization in the oxide matrix (VO + hν → VO^2+^ + 2e^−^), and (ii) photoresponse of the MXene–TiO_2_ heterostructure, which supports long relaxation times [138,139]. A comparison of EPSC under UV illumination between MXFGT and a ZTO-only control (Figure 5j) reveals superior gain and retention in the MXene-based device, indicating stronger long-term potentiation (LTP). When comparing results measured under 10 Hz input voltage pulses to those measured under 1 Hz input voltage pulses (both using parameters of 50 ms, −1 V), the EPSC gain was increased by nearly 10 times under the 10 Hz testing conditions. Furthermore, Figure 5k–m show multilevel synaptic behavior under varying light pulse widths and frequencies, including pulse-width-dependent EPSC gain and spike-rate-dependent plasticity. These behaviors not only mirror the electrical STP-LTP transition trends but also enable vision-integrated neuromorphic computing via light-modulated synaptic weights.

### 4.4. Ti_3_C_2_ MXene-Based Memristive Optoelectronic Synapse

**Figure 6:**
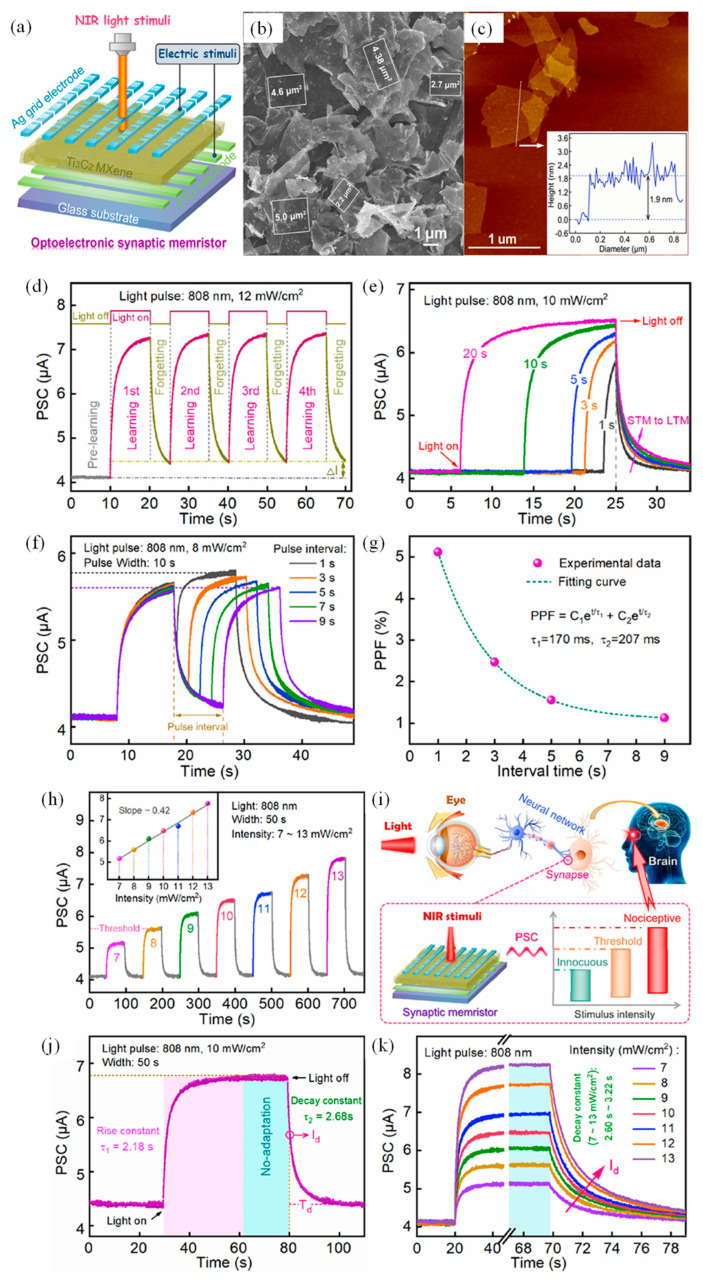
(**a**) Structural diagram of the Ag/Ti_3_C_2_/FTO optoelectronic synaptic memristor. (**b**) Field-emission scanning electron microscopy (FESEM) image showing the surface morphology of the as-synthesized Ti_3_C_2_ MXene. (**c**) Atomic force microscopy (AFM) image of the Ti_3_C_2_ nanosheets; the inset shows the measured thickness of the flakes. (**d**) Photonic response of the device under 808 nm light pulse stimulation (12 mW·cm^−2^, 10 s pulse duration, 5 s interval), exhibiting synaptic behavior. Photoresponsive current changes of the synaptic device under 808 nm illumination with varying (**e**) pulse durations (1–20 s) at an intensity of 10 mW·cm^−2^, and (**f**) pulse intervals (1–9 s) at 8 mW·cm^−2^. (**g**) Paired-pulse facilitation (PPF) index plotted against inter-pulse intervals (1–9 s); the read voltage was 1 mV. (**h**) Postsynaptic current modulation as a function of 808 nm light intensity, illustrating analog tunability. (**i**) Comparison between the biological mechanism of nociceptors in the human eye and the threshold-intensity-dependent infrared light response in the Ag/Ti_3_C_2_/FTO memristor, highlighting nociceptive and non-adaptive properties under 10 mW·cm^−2^ illumination. (**j**) Relaxation behavior of the postsynaptic current under pulsed 808 nm light stimuli. (**k**) Relaxation dynamics under varying light intensities (7–13 mW·cm^−2^), demonstrating recovery characteristics [141].

A notable advancement in this direction is the development of an optoelectronic memristor based on Ti_3_C_2_ MXene, which exhibits co-modulated synaptic plasticity under both electrical and NIR stimuli. This device architecture leverages the broadband optical sensitivity and superior electronic properties of Ti_3_C_2_ to emulate a wide range of neural behaviors, laying the foundation for bionic visual systems, especially under low-energy infrared illumination. As illustrated in Figure 6a, the device comprises an Ag/Ti_3_C_2_/FTO structure, where the Ti_3_C_2_ MXene nanosheets (thickness ~1.9 nm, Figure 6c) serve as the functional layer mimicking neurotransmitter dynamics, while Ag and FTO act as the pre- and post-synaptic terminals. Field-emission scanning electron microscopy (FESEM) reveals a typical lamellar morphology of Ti_3_C_2_ (Figure 6b), facilitating ion migration and filamentary conduction pathways essential for memristive behavior. The device exhibits nonvolatile bipolar resistive switching with stable Set/Reset voltages (~0.58 V/−0.55 V) and excellent endurance exceeding 10^3^ cycles. While the Ag/Ti_3_C_2_/FTO structure appears inorganic, its potential for flexible neuromorphic applications is entirely dependent on subsequent integration with a polymer platform like polyimide (PI). PI provides critical mechanical support and chemical inertness, protecting the MXene layer from strain-induced cracking and environmental degradation during operation on flexible substrates.

Importantly, the narrow optical bandgap of Ti_3_C_2_ (~1.71 eV) allows efficient absorption of NIR light (808 nm), enabling optically driven modulation of SW. Under pulsed NIR stimulation (12 mW/cm^2^, 10 s pulse duration), the device displays characteristic STM-like behavior with a progressive increase in baseline current upon repeated stimulation, as shown in Figure 6d. This temporal evolution mimics the biological “learning–forgetting–consolidating” cycle. Increasing the optical pulse width at fixed intensity enhances the EPSC and prolongs decay times, transitioning the device behavior from STM to LTM. This phenomenon is quantitatively described by exponential fitting (Figure 6e) with decay constants increasing from 0.82 s to 1.91 s. Further analysis reveals classic synaptic dynamics, such as (PPF, where the EPSC amplitude under consecutive light pulses diminishes with increasing inter-pulse intervals (Figure 6f,g), reflecting the temporal summation capabilities of biological synapses. Moreover, EPSC demonstrates a nearly linear dependence on light intensity, increasing from 5.12 μA to 8.24 μA over the range of 7–13 mW/cm^2^ (Figure 6h). This intensity sensitivity is harnessed to replicate nociceptive visual behaviors, where a threshold response to harmful stimuli is achieved.

The nociceptive capability of the device is schematically illustrated in Figure 6i, wherein the memristor mimics the role of an optical synapse in a retinal nerve circuit. A threshold EPSC (~5.60 μA) defines the minimum stimulus required to trigger nociceptive signaling. Unlike conventional photodetectors, the memristor maintains a high and stable EPSC upon prolonged NIR exposure, displaying “no-adaptation” behavior (Figure 6j), a crucial attribute for visual protection. Post-stimulation, the relaxation of EPSC follows a stimulus-intensity-dependent decay (Figure 6k), with time constants (τ_1_ = 2.18 s, τ_2_ = 2.68 s) that emulate real biological desensitization mechanisms. Collectively, this Ti_3_C_2_ MXene-based memristor demonstrates a comprehensive repertoire of neuromorphic functions-ranging from EPSC and PPF to STDP and experiential learning-under co-stimulation by electrical and optical inputs. Its performance in both single-device and 16 × 20 array configurations, including applications in real-time NIR imaging and nociceptive sensing, underlines the potential of MXene-enabled optoelectronic synapses in future intelligent vision and sensory systems.

### 4.5. MXene-Based Thin Film Optoelectronic Synaptic Transistor

**Figure 7:**
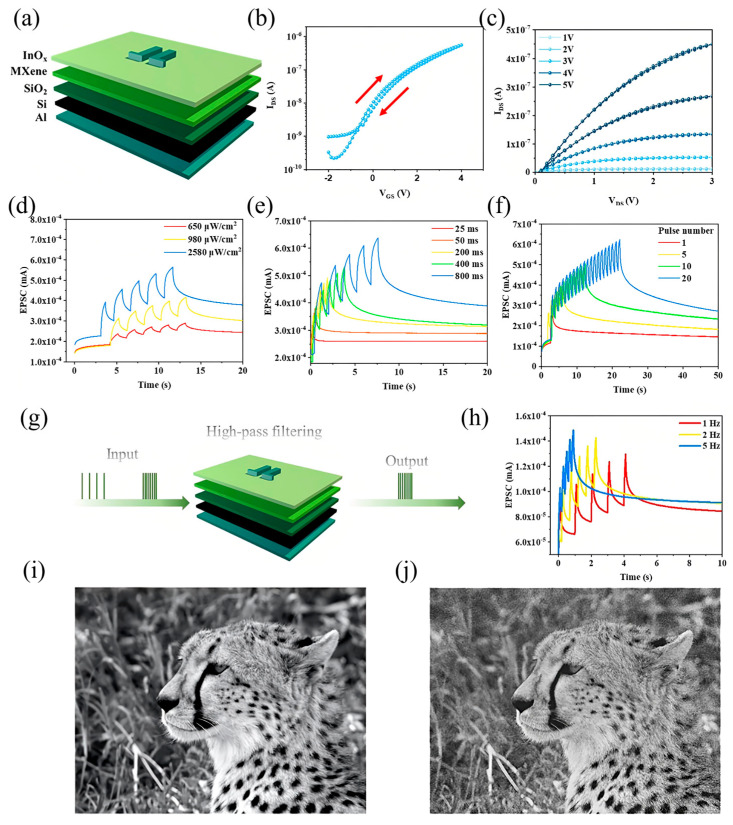
(**a**) Schematic illustration of the MXene–TiO_2_ thin-film-based artificial synaptic device. (**b**) Transfer characteristics and (**c**) output characteristics of the device, demonstrating synaptic functionality. (**d**) Excitatory postsynaptic current (EPSC) responses under varying light intensities. (**e**) EPSC modulation under different illumination pulse widths. (**f**) EPSC behavior with varying numbers of optical stimuli. (**g**) Conceptual schematic of a high-pass filter application based on the MXene artificial synaptic thin-film transistor (TFT). (**h**) EPSC response under different illumination frequencies, highlighting frequency-selective filtering behavior. (**i**) Original input image of a leopard. (**j**) Processed image of the leopard after enhancement through the high-pass filtering function of the synaptic device, demonstrating neuromorphic image processing capability [145].

Biological neurons receive a variety of interactive signals-ranging from electrical impulses to optical cues-enabling complex cross-modal processing in the brain’s multisensory association areas. Central to this functionality is the synapse, a vital junction responsible for modulating and transmitting neural information between adjacent neurons (Figure 7a). Drawing inspiration from these principles, a photoelectric artificial synaptic thin-film transistor (TFT) was developed, incorporating MXene at the dielectric interface to simultaneously exhibit electrical and optical synaptic plasticity. The device architecture (Figure 7a) features a UV-light-responsive synaptic TFT in which the InOx semiconductor serves as the channel material. During operation, UV light pulses act as presynaptic stimuli, illuminating the InOx channel while the gate terminal remains floating and a constant drain voltage (Vd = 4 V) is applied. The resulting channel current directly corresponds to the EPSC, and the channel conductance serves as the analog of SW.

This synaptic behavior is enabled by the photo-induced generation and modulation of charge carriers. The InOx film, with a bandgap of 2.8 eV, absorbs UV photons (3.4 eV) to generate abundant electron–hole pairs. Under the applied electric field, a photocurrent flows between the source and drain. Notably, some photo-generated holes are trapped at the InOx–MXene/SiO_2_ interface or within the SiO_2_ dielectric, analogous to the neurotransmitter retention and delayed release in biological synapses. These trapped charges gradually release after the UV stimulus ends, resulting in a sustained channel conductance and delayed EPSC decay-a hallmark of synaptic memory. The transfer characteristics (Figure 7b) reveal a clear clockwise hysteresis during gate voltage sweeps (−2 V to 4 V), indicating reversible and stable conductance modulation between low and high states. The output curve (Figure 7c) exhibits classic pinch-off and current saturation behavior, validating proper transistor function.

The synaptic response under varying optical stimulation conditions further highlights the device’s plasticity. As shown in Figure 7d, the EPSC amplitude increases proportionally with the intensity of UV pulses (from 650 μW/cm^2^ to 2580 μW/cm^2^) over five consecutive spikes (Δt = 1 s), demonstrating input-intensity-dependent facilitation. The device also exhibits spike-width dependent plasticity (Figure 7e): increasing pulse duration from 25 ms to 800 ms significantly enhances the EPSC peak. Furthermore, synaptic strengthening under repetitive stimulation is confirmed in Figure 7f, where EPSC amplitude increases with the number of incident light pulses (from 5 to 50), mirroring the facilitation observed in biological synapses. Beyond plasticity, this synaptic TFT demonstrates frequency-dependent signal processing behavior, akin to the high-pass filtering observed in biological synapses with low neurotransmitter release probability [142,143,144]. As depicted in Figure 7g, high-pass filtering enables selective transmission of high-frequency signals while suppressing low-frequency noise. The EPSC response under varying input frequencies (Figure 7h) rises sharply as input frequency increases from 1 Hz to 5 Hz, establishing the device’s high-pass filtering capability.

This function is visually validated through image preprocessing. A blurred image of a leopard (Figure 7i) is processed using the synaptic TFT’s frequency-selective behavior, resulting in a sharpened and clearer output (Figure 7j). Such capability illustrates the potential of MXene-based synaptic TFTs in dynamic signal enhancement tasks for intelligent vision systems and neuromorphic preprocessing units. Collectively, this device exemplifies a multifunctional optoelectronic synapse that not only emulates biological memory dynamics but also extends toward real-time analog filtering and perception, underlining the versatility of MXene-based architectures in future edge-intelligent systems.

**Figure 8:**
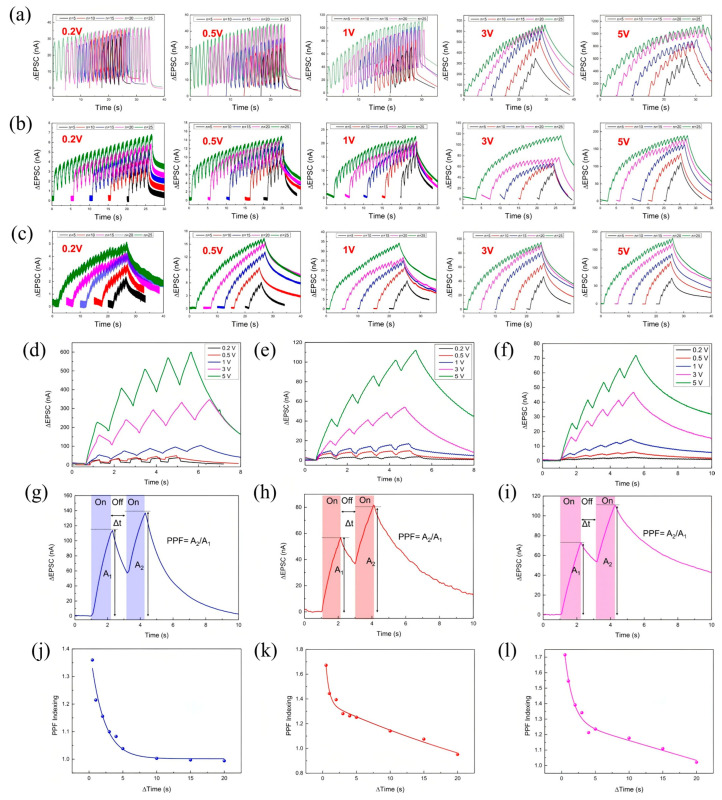
Optical synaptic plasticity characteristics of a Ti_3_C_2_T_x_-based optoelectronic device. (**a**–**c**) Short-term plasticity (STP) and long-term depression (LTD) responses under optical stimulation with varying pulse numbers (n = 5, 10, 15, 20, 25) at wavelengths of (**a**) 405 nm, (**b**) 625 nm, and (**c**) 940 nm. (**d**–**f**) STP and LTD characteristics at fixed pulse number (n = 5) under different bias voltages, illuminated at (**d**) 405 nm, (**e**) 625 nm, and (**f**) 940 nm. (**g**–**i**) Paired-pulse facilitation (PPF) index measured at a fixed interval (Δt = 1 s) under illumination at (**g**) 405 nm, (**h**) 625 nm, and (**i**) 940 nm. (**j**–**l**) PPF index as a function of inter-pulse interval (Δt) under illumination at (**j**) 405 nm, (**k**) 625 nm, and (**l**) 940 nm [153].

The artificial synaptic behavior of MXene-based devices has been extensively investigated under sequential optical pulse stimulation and cessation, reflecting fundamental memory processes inspired by Atkinson and Shiffrin’s model of learning and forgetting [146]. In this biological framework, sensory organs first register external stimuli as transient sensory memories, which correspond to STM lasting seconds to minutes. With repeated stimulation, STM can be consolidated into LTM, persisting from minutes to years [147]. Emulating this STM-to-LTM transition is essential for replicating complete learning and forgetting dynamics in artificial synapses. To demonstrate such synaptic plasticity, MXene devices were exposed to optical pulses of varying wavelengths and bias voltages, with their electrical responses capturing memory behavior [148]. Figure 8a–c display STP characteristics under illumination at 405 nm, 625 nm, and 940 nm wavelengths. For instance, Figure 8a shows the dependence of STP on pulse number (n) for 405 nm optical pulses (0.5 s duration) at various bias levels. Similar STP trends are observed for 625 nm and 940 nm illuminations (Figure 8b,c), where the post-stimulation decay reflects long-term depression (LTD), characterized by a gradual spontaneous decline of the potentiated current.

Further insight is provided by bias voltage-dependent STP/LTD behaviors at a fixed pulse number (n = 5), as illustrated in Figure 8d–f for the respective wavelengths, showing consistent synaptic modulation across the optical spectrum. The key metric of STP, PPF, was assessed by measuring the ratio of EPSCs generated by two consecutive stimuli [149,150]. Figure 8g–i reveal clear PPF responses under 1 s on/off optical pulse intervals at 405 nm, 625 nm, and 940 nm. The PPF index’s dependence on Δt is plotted in Figure 8j–l, where the fitting curves, based on an established double-exponential decay model [151], confirm two distinct relaxation times. The longer time constant τ_2_ substantially exceeds the shorter τ_1_, indicating prolonged synaptic facilitation and sustained memory effects [152]. The PPF responses at different wavelengths reveal distinct facilitation dynamics across the optical spectrum. At 405 nm, the PPF values start at 1.36 for a 0.5 s interval and gradually decrease to near unity (0.994) at 20 s, indicating a moderate synaptic facilitation that decays steadily with increasing time between pulses. In contrast, stimulation at 625 nm results in higher initial facilitation, with PPF values beginning at 1.67 for 0.5 s and tapering off to 0.95 at 20 s, reflecting stronger but similarly time-dependent synaptic enhancement. NIR stimulation at 940 nm exhibits the highest initial PPF value of 1.72 at 0.5 s, with a gradual decline to 1.02 over 20 s, demonstrating robust synaptic facilitation that sustains over longer intervals. These wavelength-dependent variations highlight the broadband optical responsiveness of MXene synapses, capable of mimicking complex temporal dynamics of biological neural systems across ultraviolet to NIR ranges.

Collectively, these results (Figure 8a–l) validate MXene devices as capable of emulating core synaptic functionalities of the human brain under broadband optical stimulation, marking them as promising candidates for neuromorphic applications spanning the UV to NIR spectrum.

## 5. MXene-Based Optoelectronic Neurons

Following the detailed discussion of MXene-based optoelectronic synapses, we now shift focus to another essential component of neuromorphic systems-artificial neurons. Neuromorphic hardware systems, inspired by the architecture and complex functionalities of the human brain, have gained significant interest as promising alternatives to traditional von Neumann computing architectures. These systems excel in tasks involving learning, perception, reasoning, and memory, while maintaining low energy consumption [1,2,3,4,5]. Typically, neuromorphic systems comprise artificial synaptic and neuronal elements. Although artificial synapses have seen considerable advances due to their energy-efficient information processing capabilities-exemplified by optoelectronic synapses such as those reported by Portner et al. [6]-artificial neurons have attracted relatively less attention, despite their critical role.

Biological neurons transmit information through a sophisticated process involving reception, integration, conduction, and output of signals [7,8,9]. Designing artificial neurons capable of emulating these complex behaviors is therefore essential. Memristor-based artificial neurons stand out for their compactness, rapid switching, and ionic dynamics analogous to biological neurons [10,11,12,13,14,15]. A fundamental characteristic of these neurons is their IF behavior, where input signals are accumulated until a threshold triggers an output spike. Various technologies, including ferroelectric devices, resistive random-access memory (RRAM), phase change memory (PCM), and spin-transfer torque magneto-RRAM (STT-MRAM), have been explored to realize artificial neurons predominantly stimulated electrically [16,17,18,19,20]. However, electrical-only stimulation poses challenges such as higher power consumption, reduced selectivity, and difficulties in replicating visual neural network functions [21]. This has spurred interest in hybrid optoelectronic neuron devices that leverage both electrical and optical signals for enhanced performance. For example, Pei et al. [22] demonstrated a quantum-dot photoelectric memristor integrated with nanosheet neurons for efficient image processing, and Yu et al. [23] developed an organic optoelectronic hybrid neuron for integrated signal processing.

### 5.1. Oxidized MXene-Based Memristive Optoelectronic Neuron

**Figure 9:**
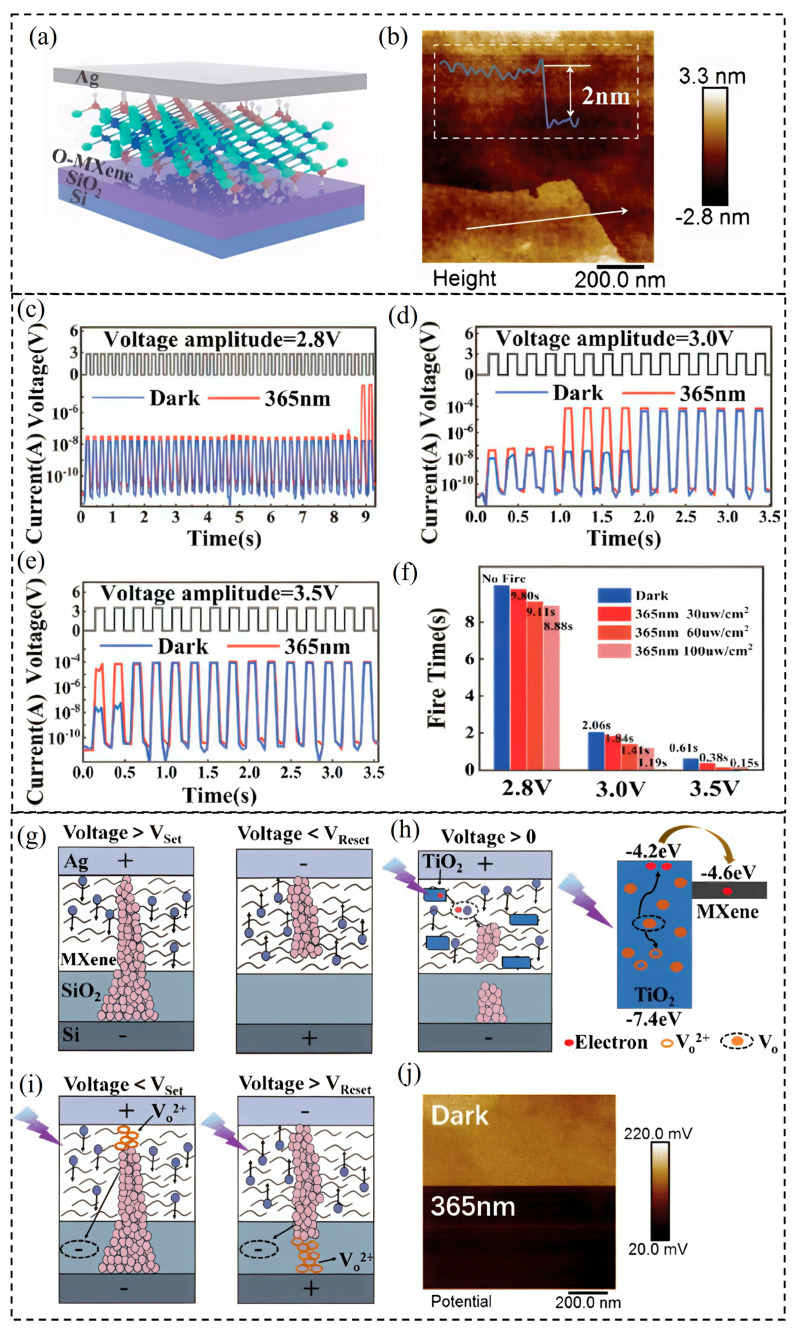
Optoelectronic and threshold-switching behaviors of the OM-AOM device. (**a**) Schematic structure of the OM-AOM device. (**b**) AFM topography image of the O-MXene film. (**c**–**e**) Integrate-and-fire (IF) behavior triggered by electrical spike trains (spike width: 90 ms; spike interval: 90 ms) with amplitudes of (**c**) 2.8 V, (**d**) 3.0 V, and (**e**) 3.5 V, recorded in both dark conditions and under UV illumination (365 nm, 100 µW/cm^2^). (**f**) Firing time variation of the OM-AOM device under different UV light intensities compared to dark conditions. (**g**) Schematic illustration of the electrically induced threshold switching mechanism. (**h**) Schematic (**left**) and corresponding energy band diagram (**right**) of threshold switching behavior under UV light (365 nm, 100 µW/cm^2^) irradiation. (**i**) Schematic diagram showing the involvement of VO^2+^ in the formation and rupture of conductive filaments (CF). (**j**) Surface potential mapping of the O-MXene film obtained via KPFM under 365 nm UV light irradiation [154].

Building upon this context, a recent study introduced an oxidized MXene-based optoelectronic memristor (OM-AOM) that functions as a hybrid artificial neuron capable of IF behavior under combined UV light and electrical stimulation. This device harnesses the strong UV photoresponse of oxidized MXene (O-MXene) to enable neuromorphic functionality. A 64 × 64 OM-AOM array was fabricated to capture and process dynamic light trajectories, demonstrating promising applications in biomimetic optoelectronics, neural prosthetics, and neuromorphic computing. MXenes offer advantageous properties including high optical transparency, mechanical robustness, and favorable optoelectronic characteristics, making them ideal candidates for neuromorphic device fabrication [24,25,26,27,28]. Specifically, their large interlayer spacing promotes rapid ion diffusion, facilitating silver ion migration critical for memristor operation. The OM-AOM device structure was fabricated on SiO_2_ substrates, with atomic force microscopy (AFM) revealing ~2 nm thick stacked O-MXene nanosheets (Figure 9a,b). Functionally, the OM-AOM device was subjected to continuous electrical spike trains with fixed amplitude (3 V), spike interval (90 ms), and varying pulse widths (60–150 ms) at frequencies below 10 Hz-aligned with biological neuron firing rates. As shown in Figure 9e, the device remained in a high-resistance state (HRS) initially and then sharply transitioned to a low-resistance state (LRS) after a certain number of spikes, clearly demonstrating IF behavior akin to biological neurons. The integration and firing times were tunable by adjusting the pulse width. At lower stimulus intensities, the switch back to HRS was delayed, indicating strong retention (Figure 9e).

Further exploration of the device’s response to electrical spikes with fixed pulse width (90 ms) and interval (90 ms) but varying voltage amplitudes (2.8, 3.0, and 3.5 V) revealed that firing was absent at 2.8 V (Figure 9c) but was triggered and accelerated at higher voltages (Figure 9d,f). Under UV illumination (365 nm, 100 μW/cm^2^), firing was induced even at the lower voltage (2.8 V) with prolonged integration times (Figure 9c), while UV modulation generally shortened integration times and extended firing duration (Figure 9d,f). Energy consumption calculations showed a reduction under UV light, evidencing improved device efficiency with optoelectronic hybrid stimulation. For example, at 3 V, energy decreased from 10.7 × 10^−5^ J (dark) to 9.9 × 10^−5^ J (UV), and at 3.5 V, from 7.4 × 10^−5^ J to 2.5 × 10^−5^ J (Figure 9f). Additionally, firing time decreased with increasing UV intensity, confirming synergistic optical–electrical signal operation.

The switching mechanism is primarily governed by the formation and rupture of silver conductive filaments (CFs). Under positive bias (Figure 9g), Ag atoms oxidize to Ag^+^ ions which migrate and reduce back to Ag atoms at the electrode, forming CFs that cause transition from HRS to LRS once voltage exceeds the SET threshold. Reversal of voltage causes oxidation and rupture of CFs at the RESET voltage, returning the device to HRS. The MXene resistive layer’s large interlayer spacing expedites Ag^+^ ion diffusion and filament dynamics. Under UV illumination (Figure 9h), the photoresponse is driven by electron transitions involving oxygen vacancy defects (VO) in TiO_2_ formed by MXene oxidation. These defects narrow the bandgap or create trap states, facilitating electron excitation. Photon absorption by oxygen vacancies (VO + hν → VO^2+^ + 2e^−^) generates electrons that assist Ag^+^ reduction, accelerating CF formation and lowering the SET voltage, while RESET remains unaffected. The noisy I–V behavior under UV (Figure 9g) results from accumulation of VO^2+^ ions near electrodes, forming fragile vacancy filaments modulating CFs (Figure 9i). Kelvin probe force microscopy (KPFM) (Figure 9j) further confirms decreased surface potential under UV, verifying oxygen vacancy ionization and electron generation. Overall, this oxidized MXene-based optoelectronic memristor demonstrates multifunctional neuron-like IF behavior with tunable electrical and optical inputs, showcasing a significant advancement toward hybrid neuromorphic systems that integrate optoelectronic sensory processing with energy-efficient computation.

### 5.2. MXene-Based Optoelectronic Hybrid-Integrated Neuron

An artificial optoelectronic hybrid-integrated neuron (AOHN) based on Ag nanoparticle-decorated MXene has been proposed. The decorated MXene enables low-voltage operation (below 1 V) and provides the device with the ability to integrate optoelectronic signals across time and space. This multifunctional neuron significantly expands the capabilities of neuromorphic systems. They further demonstrated an integrated visual perception system designed to emulate human-like conditional responses. This system comprises a polymer electret transistor (artificial synapse), the proposed AOHN, and a robotic hand. By integrating optical sensory inputs and electrical training signals, the system’s response time can be effectively modulated. Additionally, a 28 × 28 spiking neural network (SNN) was constructed using the developed artificial synapses and neurons, achieving successful multi-task recognition of digit patterns and rotation angles through spatiotemporal information integration.

**Figure 10:**
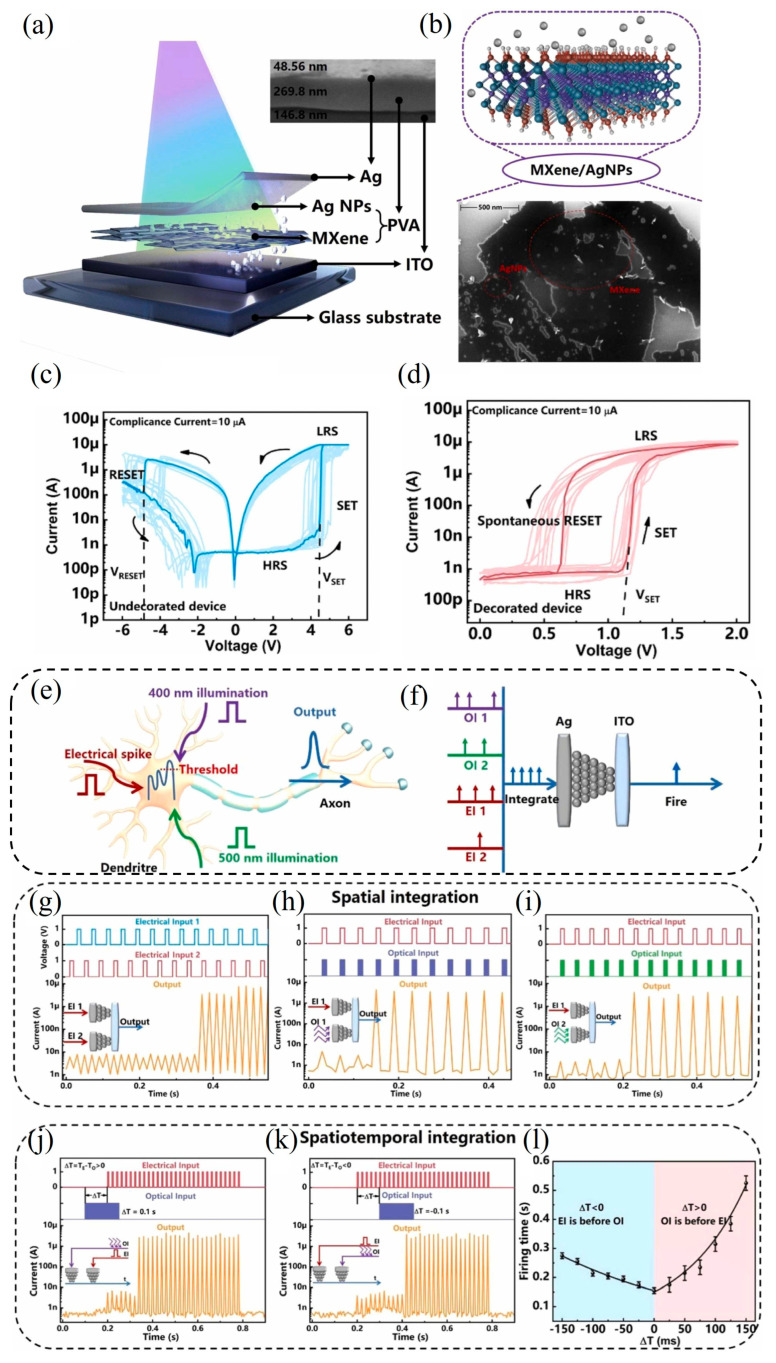
Device structure, morphology, electrical properties, and spatiotemporal integration behavior of the AOHN. (**a**) Schematic diagram of the AOHN structure, consisting of a resistive layer sandwiched between Ag and ITO electrodes. (**b**) Scanning electron microscopy (SEM) image and 3D molecular structure of MXene nanosheets. (**c**,**d**) I–V characteristic curves of the AOHN device: (**c**) undecorated structure and (**d**) decorated structure, exhibiting threshold switching behavior (reduction in the SET voltage (from 4.37 V to ~1.14 V) (**e**) Schematic illustration of a biological neuron integrating both electrical and optical inputs. (**f**) Schematic of the AOHN implementing electrical–optical signal integration. (**g**) Spatially integrated current response at two electrical input (E_I_) terminals triggered by electrical spikes (1 V amplitude, 10 ms width, 25 Hz frequency). (**h**) Spatially integrated current response to combined E_I_ and optical input (O_I_) signals under UV and green light illumination. (**i**) Spatiotemporal integrated current in response to non-simultaneous EI and OI signals, triggered by a train of 30 electrical spikes (1 V amplitude) and a UV light pulse (100 μW/cm^2^ intensity, 150 ms duration), with a 0.1 s inter-spike interval. (**j**) Spatiotemporal integration when electrical stimulation precedes optical stimulation (ΔT = 0.1 s). (**k**) Spatiotemporal summation results as a function of varying inter-spike intervals between two inputs, along with corresponding fitting curves. (**l**) Spatiotemporal summation results varying inter-spike interval of two inputs and fitting results. [157].

In summary, the proposed AOHN addresses current limitations in artificial neurons and demonstrates strong potential as a functional component in advanced neuromorphic systems. The optoelectronic neuron is fabricated on a glass substrate with a typical device architecture comprising indium tin oxide (ITO)/PVA:MXene–Ag nanoparticles (NPs)/Ag (Figure 10a). Ag NPs are uniformly assembled on the surface of MXene nanosheets through a hydrophilic polyvinyl alcohol (PVA) solution. In this configuration, PVA plays a dual role: it functions as a crosslinking agent to facilitate the initial bonding between Ag NPs and MXene, it also serves as a dielectric layer that regulates Ag^+^ ion migration and as a flexible foundation. The density of hydroxyl groups in the PVA chain directly determines the ion conductivity and therefore the neuron’s firing threshold and energy consumption. This highlights how polymer chemistry is inextricably linked to core neuromorphic functionality. The morphology of the MXene nanosheets is characterized by SEM (Figure 10b) and transmission electron microscopy (TEM), which confirm that the Ag nanoparticles, with an average diameter of approximately 15 nm, are uniformly distributed across the surface of the MXene nanosheets.

To investigate the threshold-switching behavior, the electrical properties of the device were measured under dark conditions. Figure 10c presents the output characteristics of the device without Ag nanoparticle (NP) decoration during a voltage sweep from 0 V → 6 V → –6 V → 0 V, under a compliance current (ICC) of 10 μA. The current increases sharply, transitioning the device from an HRS to an LRS at a SET voltage $\left(V_{SET}\right)$ of approximately 4.37 V. Conversely, a rapid current decrease occurs at a RESET voltage $V_{RESET}$ of around 4.8 V, returning the device to the HRS. This threshold-switching behavior is primarily attributed to the drift of Ag^+^ ions and the subsequent formation and rupture of Ag conductive filaments (CFs). Notably, as shown in Figure 10d, the Ag-decorated MXene (D-MXene) device exhibits a significantly reduced SET voltage, along with a narrower distribution of threshold voltages. This improved performance indicates enhanced switching uniformity and reduced energy consumption, highlighting the benefits of Ag NP decoration in facilitating more efficient and stable switching dynamics.

A fundamental function of biological neurons is the integration of synaptic input signals received from multiple terminals into a specific output spike, which is critical for neural transmission and computation. This includes processing of spatially distributed inputs from different synapses (spatial integration) and temporally separated events (temporal integration) [40,155]. Figure 10e illustrates a schematic of a biological neuron integrating both electrical and optical signals collected at the dendrites. The dendritic membrane then determines whether the local graded potential exceeds a threshold, thereby initiating a spike along the axon or remaining inactive-following the well-known all-or-none principle [156]. To emulate this behavior, Figure 10f presents a schematic of the artificial integration function realized in the AOHN device. Multiple electrodes deposited on the resistive layer serve as distinct electrical input $\left(E_{I}\right)$ terminals, while two distinct illumination wavelengths (400 nm and 500 nm) represent optical input (OI) terminals. As demonstrated in Figure 10g, spatial integration of electrical signals is evaluated by applying spikes to $E_{I_{1}}$ and $E_{I_{2}}$. When either $E_{I_{1}}$ or $E_{I_{2}}$ is stimulated individually, the neuron fires after approximately 0.56 s. However, simultaneous stimulation of both terminals significantly shortens the firing time to ~0.38 s, demonstrating enhanced signal summation. Figure 10h,i highlight the AOHN’s photoresponse under UV (400 nm) and green light (500 nm) illumination, respectively. The firing time is further reduced to ~0.16 s and ~0.24 s under UV and green light, respectively, indicating efficient optically driven modulation.

In neuroscience, neural activities are often driven by the synergistic effect of electrical and optical signals, which exhibit pronounced spatial and spatiotemporal dependencies [40]. The AOHN device successfully emulates this behavior by achieving spatiotemporal integration of optoelectronic inputs. A series of electrical spikes and a UV light pulse were sequentially applied to the device with a controlled inter-spike interval (ΔT = TE − TO). As shown in Figure 10j, when the optical pulse precedes the electrical input by 100 ms (ΔT > 0), the neuron fires at ~0.28 s. Conversely, when the optical input follows the electrical signal by 100 ms (ΔT < 0), the firing time decreases to ~0.22 s. For simultaneous stimulation (ΔT = 0), as depicted in Figure 10k, the firing time is shorter compared to either delayed condition.

The dependence of firing time on ΔT is systematically summarized in Figure 10l, revealing a characteristic asymmetric response curve. This behavior is indicative of temporal causality in stimulus integration and can be quantitatively fitted using exponential functions [53], underscoring the device’s capability for dynamic spatiotemporal information processing.

$$T_{fire}=\left\{\begin{array}{c} T_{s}\;exp\left(\frac{∆T}{\tau_{+}}\right),∆T>0\\ T_{s}\;exp\left(\frac{∆T}{{-\tau }_{-}}\right),∆T<0 \end{array}\right\}$$

Here, TS denotes the firing time when optical and electrical signals are applied simultaneously to the device, measured to be approximately 0.16 s. The dependence of firing time on the inter-spike interval is characterized by the time constants $\tau_{+}$ and $\tau_{-}$, fitted as 102 ms and 263 ms, respectively. These parameters describe the asymmetric response of the device to varying temporal sequences of input stimuli. Based on these results, the programmable integration of optical and electrical signals in specific temporal orders enables sequence recognition within the proposed optoelectronic neural circuits-a capability that will be further explored in subsequent sections. In summary, the proposed AOHN exhibited several distinct advantages. First, from a structural and material standpoint, the AOHN leverages an organic resistive layer that enables ultra-simple fabrication, excellent thin-film flexibility, and tunable electrical properties-making it a practical and scalable platform for next-generation flexible electronics. Second, the device demonstrates enhanced electrical performance, operating at low voltage with a high on/off ratio, thereby surpassing many conventional electrically driven artificial neurons in terms of energy efficiency and signal clarity. Third, the AOHN offers tunable spiking dynamics, as its firing time can be precisely modulated through an additional, contactless optical input terminal, allowing for dynamic and remote control of neuronal behavior. Fourth, the device supports seamless functional integration into intelligent visual perception systems, where it can act as a core processing unit to emulate human-like conditional responses. Finally, by integrating optoelectronic stimuli, the AOHN enables multifunctional pattern recognition, significantly improving recognition efficiency and introducing new computational functionalities within spiking neural networks.

## 6. MXene-Based Fully Integrated Neuromorphic Computing Systems

Building upon the advances in MXene-based artificial synapses and neurons, the development of fully integrated neuromorphic hardware systems remains crucial for realizing brain-inspired computing architectures with enhanced flexibility and efficiency. Traditional computing paradigms are fundamentally limited by the von Neumann bottleneck, which separates memory and processing units and constrains performance, particularly for complex tasks like big data analysis and artificial intelligence [1,2,3,4,5]. Neuromorphic chips, designed to mimic the interconnected architecture of biological neural networks, offer a promising alternative by enabling energy-efficient, parallel information processing through arrays of artificial neurons and synapses.

**Figure 11:**
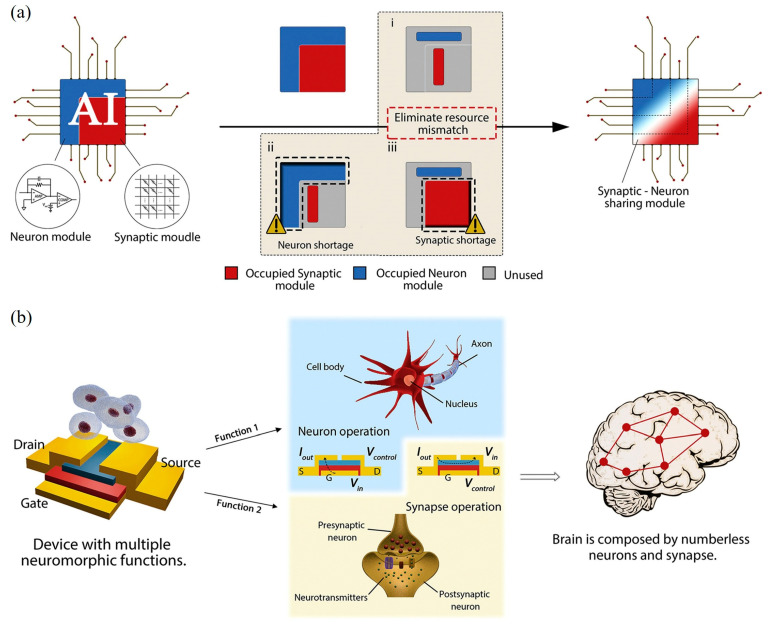
Neuromorphic chip with synaptic–neuronal sharing modules. (**a**) To mimic the structure of a biological brain, a conventional neuromorphic chip consists of separate neuron and synapse modules. Beyond the ideal case-where hardware resources perfectly match algorithmic requirements-three types of mismatch scenarios may occur during task execution: (**i**) Both neuron and synapse modules are largely underutilized; (**ii**) Synapse modules are underutilized while neuron modules are insufficient; (**iii**) Neuron modules are underutilized while synapse modules are insufficient. These mismatches highlight the need to break the rigid separation between synaptic and neuronal modules, enabling flexible sharing and dynamic resource allocation. (**b**) The core of a neuromorphic chip with synaptic–neuronal sharing modules is a multifunctional device capable of performing both synaptic and neuronal functions, with the ability to switch between them via simple electronic programming [177].

State-of-the-art neuromorphic platforms such as Intel’s Loihi and IBM’s TrueNorth integrate large numbers of neurons and synapses—128,000 neurons with 128 million synapses [158] and 64 million neurons with 16 billion synapses [159], respectively—emulating the dense connectivity of the brain. In biological systems, neurons communicate via discrete electrical spikes transmitted through synapses, where synaptic weights modulate signal strength and learning processes [160,161,162]. Neuromorphic hardware replicates these mechanisms using electronic components to realize artificial neurons and synapses, as illustrated in Figure 11a. Such devices have enabled significant improvements in computational domains including convolutional processing [163,164], image recognition [165,166], and biosignal monitoring [167,168]. However, current neuromorphic architectures predominantly rely on distinct device types-such as CMOS circuits [169,170], memristors [72,171], and transistors [172,173,174]-to separately implement synaptic and neuronal functionalities. This physical and functional separation complicates fabrication processes, leads to inefficient resource utilization, and rigid hardware-software mappings that limit adaptability (Figure 11a). Conventional systems often suffer from fixed functional roles, where devices act solely as either synapses or neurons, restricting the ability to dynamically reconfig network topology or optimize speed beyond synaptic weight tuning alone. Addressing these challenges frequently involves scaling up system size through multiple chip deployments [175,176], which increases complexity, cost, and fragmentation of resources.

To overcome these limitations, we propose a paradigm shift toward multifunctional neuromorphic devices that integrate synaptic and neuronal behaviors within a single structure. Specifically, we introduce a switchable neuronal-synaptic transistor (SNST) based on two-dimensional MXene materials (Figure 11b). This innovative device leverages MXene’s outstanding electrical and optical properties to realize programmable functional switching between synapse-like and neuron-like operation modes through straightforward circuit-level rewiring. The shared fabrication process enhances manufacturability while reducing device heterogeneity. By constructing neural networks composed of multiple SNSTs, we demonstrate flexible, on-demand allocation of computational resources through dynamic reconfiguration between synaptic and neuronal functionalities. This approach significantly decreases the total number of devices required for a given task, pushes resource utilization close to 100%, and allows simultaneous optimization of synaptic weights and network topology. The resulting system exhibits nearly 200% faster operation compared to traditional neuromorphic architectures, marking a substantial advancement toward practical, scalable, and energy-efficient brain-inspired computing platforms. In summary, the SNST device exemplifies the potential of MXene-based multifunctional components to unify synaptic and neuronal roles, paving the way for a top-down programmable neuromorphic hardware platform that achieves superior accuracy, efficiency, and flexibility. This progress complements earlier developments in MXene optoelectronic synapses and neurons, collectively driving the frontier of next-generation neuromorphic systems.

**Figure 12:**
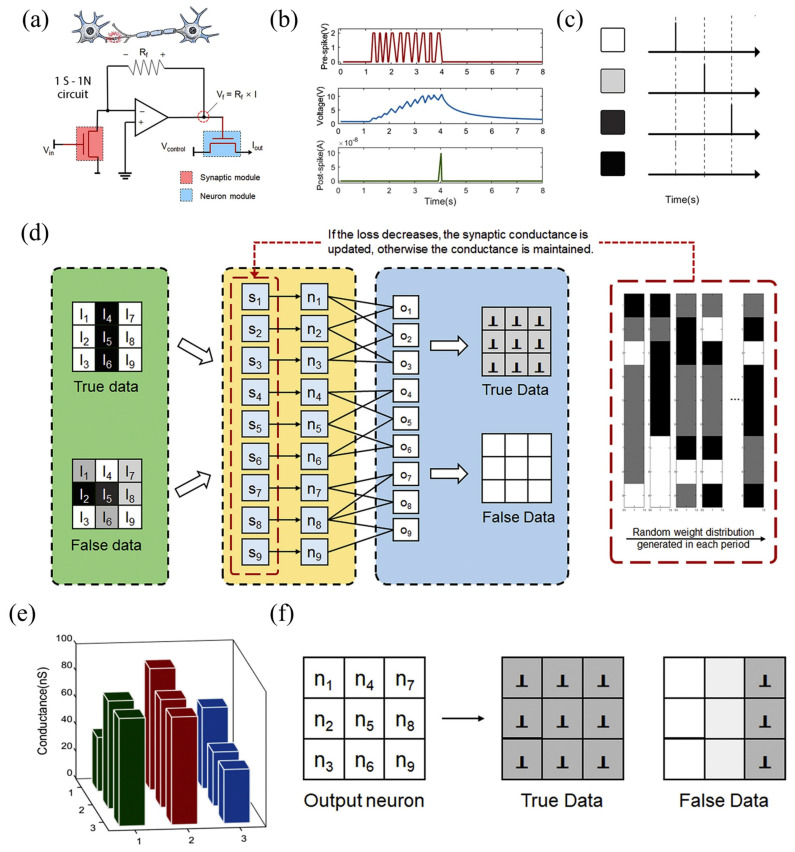
Neuromorphic hardware network based on the SNST (Spiking Neuron Synaptic Transistor). (**a**) Schematic diagram of the 1S–1N circuit configuration. (**b**) Signal flow of the 1S–1N circuit: the first row shows the input to the 1S–1N circuit; the second row shows the amplifier output used as input to the neuron; and the third row shows the final output of the 1S–1N circuit. (**c**) First-spike time coding representing grayscale image information. (**d**) Architecture of the SNST-based neuromorphic hardware network, composed of an input layer (green, left), a hidden layer (orange, center), and an output layer (blue, right). (**e**) Synaptic weight distribution in the trained hardware network. (**f**) Output activity of the trained network, where output neurons exhibit bursting behavior in response to real image inputs [177].

Electrical characterizations of a single switchable neuronal-synaptic transistor (SNST) further validate its unique dual-functionality, enabling it to operate seamlessly as both a neuron and a synapse within neuromorphic circuits. This intrinsic multifunctionality significantly enhances the adaptability and architectural flexibility of SNST-based neuromorphic chips. Unlike traditional architectures that rely on physically and functionally separate synaptic and neuronal components, SNST arrays facilitate dynamic reconfiguration of the neuron-to-synapse ratio. This flexibility effectively addresses the common mismatch between hardware resource allocation and task-specific algorithmic demands, which often constrains the efficiency and scalability of conventional neuromorphic systems. By maintaining a fixed total number of devices, SNST-based networks can execute at least K times more tasks compared to circuits with discrete synaptic and neuronal units, where K represents the synapse-to-neuron ratio. To demonstrate this capability, a minimalistic neuromorphic network using SNST devices was implemented. As depicted in Figure 12a, a basic 1S–1N circuit consists of one SNST configured in synaptic mode and another in neuronal mode, along with necessary supporting electronic components. This arrangement emulates a fundamental biological synapse-neuron connection. The system employs a first-pulse emission time coding strategy [178], wherein the timing of the first neuronal spike encodes input information. Specifically, the number of input pulses to the synaptic SNST (left device in Figure 12a) corresponds to grayscale intensity levels. These pulses generate postsynaptic currents that are amplified and transmitted to the neuronal SNST (right device), which produces output spikes. Figure 12b illustrates the temporal relationship of input pulses, amplified signals, and neuronal spike outputs across three corresponding rows.

Figure 12c further highlights how different grayscale inputs produce distinct spike timings, validating the encoding scheme. Building on this foundational 1S–1N architecture, a simplified neuromorphic network simulation was conducted, shown in Figure 12d. The hardware system is organized into three layers: input (green), hidden (orange), and output (blue). The input layer comprises nine pixels that emit variable pulse counts based on grayscale values, which connect via synapses to nine neurons in the hidden layer. These hidden neurons are linked through conductance lines to nine output-layer neurons. Following training, the network exhibits expected classification behavior: all output neurons activate in response to ‘true’ input patterns (e.g., images containing central black stripes), while only partial neuron activation occurs for ‘false’ inputs lacking this feature. Synaptic weights adapt throughout training to optimize decision boundaries, as depicted in Figure 12e and further supported by Figure 12a. Here, higher synaptic conductance values indicate stronger influence from corresponding input pixels during classification. The resulting classification accuracy, presented in Figure 12f, confirms the network’s effectiveness in discriminating binary image data based on temporal spike coding (Table 1).

**Table 1:** Comprehensive overview of the devices discussed in the reviewed studies, including their classifications, functions, and potential applications.

Substrate/Device Type	Key Synaptic/Neuronal Functions	Application/Context	Ref
CMOS-based circuitry (IBM TrueNorth chip)	Real-time AI performance at ultralow power (63 mW)	Emulation of neuronal activity (integrating one million spiking neurons and 256 million synapses)	[8]
Memristive Synapses based on Lightly Oxidized Sulfide Films	Ultra-sensitivity	Ultrasensitive Synapses	[11]
Vertically integrated spiking cone photoreceptor arrays	Spiking cone photoreceptor functions	Color perception	[12]
Ionic Floating-Gate Memory Array	Parallel programming	Scalable neuromorphic computing	[15]
CMOS-compatible electrochemical synaptic transistor arrays	Synaptic function (implied deep learning acceleration)	Deep learning accelerators	[16]
Vertical Silicon Nanowire Based Single Transistor Neuron	Excitatory, Inhibitory, and Myelination Functions	Highly scalable neuromorphic hardware	[18]
Phase-change heterostructure	Ultralow noise and drift	Memory operation	[20]
Ferroelectric Junction-less Double-Gate Silicon-On-Insulator FET	Tripartite Synapse function	Tripartite Synapse Emulation	[21]
Antiferromagnet/Ferromagnet Heterostructure	Artificial Neuron and Synapse, dynamics of Spin–Orbit Torque Switching	Artificial Neuron and Synapse	[26]
Artificial optic-neural synapse	Energy consumption as low as 66 fJ per spike	Colored and color-mixed pattern recognition	[32]
2D material neural network image sensors	Real-time optical encoding (at speeds of 20 million bins per second)	Ultrafast machine vision	[33]
Bioinspired photoresponsive single transistor neuron	Neuron function	Neuromorphic Visual System	[44]
MXene/Violet Phosphorus van der Waals Heterojunctions	Long-Term Plasticity (LTP)	Optoelectronic Neuromorphic Computing	[123]
Plasmonic Optoelectronic Memristor	Fully Light-Modulated Synaptic Plasticity	Neuromorphic Vision	[59]
Synaptic memristor based on two-dimensional layered WSe_2_ nanosheets	Short- and long-term plasticity (STP/LTP)	Synaptic applications	[140]
Two-Dimensional Metal–Organic Framework Film	Transition from STP to LTP	Optoelectronic Synaptic Plasticity	[116]
IGZO-Alkylated Graphene Oxide Hybrid Structure	Optoelectronic Synapse	Optoelectronic Synapse	[121]
MXene-based optoelectronic synaptic transistors (MXene–TiO_2_–PDVT-10 composite)	STM-LTM transition, PPF (dual-exponential decay), Multispectral sensitivity	Hierarchical responses and attentional mechanisms	[130]
Ag/Ti_3_C_2_/FTO memristor	STM/LTM, PPF, Nociceptive visual behaviors (threshold response), No-adaptation behavior	Near-Infrared Artificial Vision Applications	[141]
Ti_3_C_2_T_x_-based optoelectronic device	Optically modulated, mechanically flexible, visible-to-near IR broadband-responsiveness (STP, LTD, PPF)	Flexible neuromorphic applications	[153]
Switchable Neuronal-Synaptic Transistor (SNST) based on 2D MXene	Programmable functional switching, Dynamic reconfiguration, Temporal spike coding	High-efficiency neuromorphic hardware network	[177]

## 7. Summary and Future Prospects

MXenes, as two-dimensional materials with exceptional electrical conductivity, tunable surface chemistry, and mechanical flexibility, present a promising platform for next-generation optoelectronic neuromorphic devices. Their atomic-scale structure facilitates efficient ion transport, enabling fast, low-energy synaptic and neuronal functions, while optical modulation further enhances device speed and reduces power consumption. These attributes position MXenes as ideal candidates for multifunctional neuromorphic systems capable of complex neural behaviors and hybrid electrical–optical signal processing. However, challenges persist, including controlling defect-induced variability, improving material stability against oxidation, and developing scalable, reproducible synthesis and fabrication methods that maintain MXenes’ properties. A deeper mechanistic understanding of ion migration, filament dynamics, and surface chemistry interplay is critical to optimize device performance and reliability. Moreover, integration with existing semiconductor technologies and standardization of testing protocols remain open hurdles.

Looking forward, MXene-based devices offer significant potential for flexible, energy-efficient neuromorphic computing in wearable electronics, artificial vision, robotics, and large-scale neural networks. Their inherent multifunctionality and optoelectronic responsiveness enable advanced sensory integration and adaptive learning, promising breakthroughs in brain-inspired hardware that overcome the von Neumann bottleneck. Continued interdisciplinary research is essential to translate these materials from laboratory prototypes to scalable, commercially viable neuromorphic technologies that will underpin future intelligent computing systems.

## 8. Conclusions and Future Directions

MXene-based optoelectronic neuromorphic devices represent a highly promising frontier in brain-inspired computing, marrying unique material properties with functional versatility to emulate complex neural behaviors. The inherent electrical conductivity, surface tunability, and mechanical flexibility of MXenes facilitate the design of synaptic and neuronal devices capable of hybrid electrica–optical operation, enabling reduced energy consumption and enhanced computational speed. Such devices exhibit key neuromorphic functions including synaptic plasticity, integrate-and-fire neuron behavior, and spatiotemporal signal processing, which are essential for building advanced artificial neural networks. Importantly, emerging multifunctional architectures, such as synaptic–neuronal switching transistors, address the limitations of rigid hardware allocations and pave the way for dynamically reconfigurable neuromorphic systems with superior efficiency and scalability. However, MXene-based optoelectronic synapses and neurons face several key limitations, including material instability (oxidation, non-uniform surface terminations); toxicity concerns from fluoride-containing synthesis; and device-level issues like conductance drift, noise, and limited spectral selectivity. Large-scale implementation is further hindered by device variability, endurance limits, and manufacturing challenges such as achieving uniform wafer-scale films and CMOS compatibility. Finally, the lack of standardized models and the non-ideal, nonlinear nature of conductance updates complicate circuit design and algorithm co-optimization. Addressing these challenges will require improved synthesis (F-free routes), robust encapsulation, scalable deposition, and better device modeling to achieve reproducible, stable, and energy-efficient neuromorphic systems.

Future research must prioritize comprehensive mechanistic understanding, innovative synthesis methods, and robust integration strategies to translate laboratory prototypes into commercially viable platforms. On the materials side, efforts are moving toward fluorine-free and environmentally friendly synthesis routes with precise control of surface terminations (–O, –OH, –S) to improve stability, biocompatibility, and photoresponse. Researchers are also exploring alloyed and heterostructured MXenes, as well as van der Waals stacks with TMDs, perovskites, and quantum dots, to enable tunable band alignment, wavelength-selective photoresponses, and low-noise operation. Plasmonic and photothermal engineering will be used to localize light fields and achieve highly energy-efficient optical spikes. At the device and circuit level, MXenes will enable richer plasticity behaviors such as spike-timing-dependent plasticity, multi-modal synaptic learning, and color-encoded weights for optical multiplexing. Hybrid optical–electrical stimulation schemes, low-variation memristive switching, and noise-tolerant weight updates will improve reliability and reproducibility. Integration with CMOS back-end processes and silicon photonics will allow compact opto-neurons, on-focal-plane computing for vision systems, and direct coupling with micro-LED or waveguide sources for dense, high-speed interconnects.

Scaling up to large arrays and systems, wafer-scale printable MXene inks, 3D stacked architectures, and algorithm–device co-design will enable crossbars with millions of programmable synapses. These arrays will be benchmarked on real-world datasets with device-in-the-loop training, targeting ultralow energy consumption (<10 fJ per synaptic event) and high endurance (>10^9^ cycles). Encapsulation strategies and standardized safety protocols will address MXene oxidation, fluoride exposure, and biocompatibility concerns, making devices reliable for wearable, biomedical, and edge-AI applications.

In the broader trend, MXene optoelectronic synapses are expected to underpin retina-like vision sensors, multispectral perception systems, and distributed edge intelligence that operates with minimal energy. Establishing compact device models, process design kits, and open reliability data will accelerate ecosystem development. Overall, the field is moving toward photonic-CMOS co-designed, wafer-scale, safe, and energy-frugal neuromorphic hardware, with MXenes positioned as a versatile and scalable material platform for next-generation brain-inspired computing. With continued interdisciplinary efforts, MXene-based optoelectronic neuromorphic devices are poised to revolutionize intelligent computing, enabling energy-efficient edge AI, flexible sensory interfaces, and sophisticated cognitive robotics that approach biological intelligence in functionality and adaptability.

## Data Availability

All data generated or analyzed during this study are included in this published article.
